# Development and Evaluation of a Fusion Polyprotein Based on HspX and Other Antigen Sequences for the Serodiagnosis of Tuberculosis

**DOI:** 10.3389/fimmu.2021.726920

**Published:** 2021-10-04

**Authors:** Fangbin Zhou, Xindong Xu, Xiaobing Cui, Weiqing Pan

**Affiliations:** ^1^ Department of Tropical Diseases, Naval Medical University, Shanghai, China; ^2^ Clinical Medical Research Center, The Second Clinical Medical College, Shenzhen People’s Hospital, Jinan University, Shenzhen, China; ^3^ Institute for Infectious Diseases and Vaccine Development, Tongji University School of Medicine, Shanghai, China

**Keywords:** *Mycobacterium tuberculosis*, active TB, fusion polyprotein, serodiagnosis, sensitivity, specificity

## Abstract

**Background:**

The lack of suitable diagnostic tools contributes to the high prevalence of tuberculosis (TB) worldwide. Serological tests, based on multiple target antigens, represent an attractive option for diagnosis of this disease due to their rapidity, convenience, and low cost.

**Methods:**

Measures to reduce non-specific reactions and thereby improve the specificity of serological tests were investigated, including blocking antibodies against common bacteria in serum samples and synthesizing polypeptides covering non-conserved dominant B-cell epitopes of antigens. In addition, a fusion polyprotein containing HspX and eight other antigen sequences was constructed and expressed to increase overall sensitivity of the tests.

**Results:**

Inclusion of *Escherichia coli* lysate partially increased the specificity of the serological tests, while synthesis and inclusion of peptides containing non-conserved sequences of TB antigens as well as dominant B-cell epitopes reduced non-specific reactions without a decrease in sensitivity of the tests. A polyprotein fusing HspX and eight other antigen sequences was constructed and displayed 60.2% sensitivity, which was higher than that of HspX and the other individual antigen segments. Moreover, the specificity of the polyprotein was 93.8%, which was not significantly decreased when compared with HspX and the other individual antigen segments.

**Conclusions:**

The roles of the fusion polyprotein in the humoral immune response against TB infection were demonstrated and provide a potential novel approach for the development of TB diagnostics.

## Introduction

Tuberculosis (TB) is one of the most prevalent infectious diseases and among the top 10 causes of death worldwide despite substantial progress toward TB control over the last decades. In 2020, there were an estimated 10.0 million new TB cases and 1.2 million deaths globally due to TB (1). The emergence of multidrug-resistant tuberculosis (MDR-TB) and extensively drug-resistant tuberculosis (XDR-TB), and the spread of HIV/AIDS in TB-endemic regions have impeded efforts to control and eliminate TB and prompted health authorities to strengthen and reinforce control strategies to limit their spread (2–4).

Several methods for diagnosis of TB are currently available, including sputum smear and culture tests, chest X-ray and immunodiagnostic detection are currently available. However, a rapid, accurate, and cost-effective diagnostic tool for TB is urgently required to control this disease. Biomarkers predicting treatment efficacy and cure of active TB, the reactivation of latent tuberculosis infection (LTBI), and the induction of protective immune responses by vaccination have been investigated (5–7). However, TB-specific biomarkers have not yet been discovered and the qualification of biomarkers as a surrogate for a clinical endpoint in TB is very challenging. Immunodiagnostic detection of antigens or their cognate antibodies in the blood of patients has been successfully applied to other pathogens (8) and thus is an attractive option for TB. However, the sensitivities and specificities of the currently available options based on single or multiple target antigens for TB are variable and do not yet meet the requirements for clinical use. In 2011, the World Health Organization (WHO) issued a policy recommendation against the use of the various commercial serological tests for TB diagnosis due to the suboptimal sensitivity and specificity (9). However, further research and development in this field, specifically the identification and screening of novel serodiagnostic antigens, is still highly recommended by WHO (10).

A major challenge for TB serodiagnosis is false-positive reactions in healthy individuals, which reduces the specificity of the tests. In addition to cross-reactivity with Bacillus Calmette-Guérin (BCG) vaccination and other environmental mycobacteria, most TB antigens have sequences that are homologous those of other common bacteria, and TB serum antibodies inevitably cross-react with antigens from these bacteria. One way to reduce such non-specific reactions is to block antibodies against these bacteria by pre-adsorbing serum samples with lysates of common bacteria. Another approach to reduce non-specific reactions is through bioinformatics analysis to select non-conserved fragments of TB antigens compared with other bacteria while simultaneously retaining dominant B-cell epitopes where possible. However, sensitivity might decrease with this approach. Goyal et al. used B-cell epitope-containing peptides of RD1 (ESAT-6, CFP-10) and RD2 (CFP-21, MPT-64) antigens for immunodiagnosis of pulmonary TB (11). Afzal et al. constructed fusion proteins tn1FbpC1-tnPstS1 and tn2FbpC1-tnPstS1 with immunodominant B-cell epitope sequences and found that removal of a non-epitopic FbpC1 region (amino-acid residues 34–96) unmasked some of the epitopes, resulting in greater sensitivity (12).

To data, no single TB antigen-based assay has achieved a satisfactory serodiagnostic performance, which impelled us to identify new protein targets and investigate different combinations of currently identified antigens. Strategies to improve sensitivity of the tests include mixing recombinant antigens, fusing recombinant antigens (or segments), and peptide-based antibody detection. Zhang et al. mixed and combined three antigens—Rv3425, 38 kDa, and LAM—to developed a multiple-antigen enzyme-linked immunosorbent assay (ELISA) test, which was a potentially useful tool for the serodiagnosis and screening of active TB (13). Yang et al. constructed a recombinant fusion protein Rv0057-Rv1352 that exhibited good immunoreactivity with serum from patients with TB (14). A recombinant fusion of three immunodominant antigens (38-kDa-16-kDa-ESAT-6) was also reported (15). Another approach to TB serodiagnosis involved fusing antigen fragments, which contained dominant linear B-cell epitopes, instead of whole protein antigens. Two novel polyproteins, 38kD-ESAT6-CFP10 (38F) and Mtb8.4-MPT64-TB16.3-Mtb8 (64F), were constructed and evaluated by Feng et al, with the novel 38F-64F indirect ELISA exhibiting effective diagnostic performance (16). Recently, several serological tests based on synthetic peptides derived from highly antigenic proteins were also designed and evaluated. Four peptides (7–10 amino acids in length) corresponding to group-specific epitopes of Ag 85 complex of *Mycobacterium tuberculosis* (*M. tb*) were synthesized and the peptide-based ELISA found to be a sensitive, specific, rapid, and cost-effective immunoassay for early diagnosis of pulmonary and extrapulmonary TB (17). Another study evaluated the combination of peptides from B-cell epitopes of ESAT-6, CFP-10, CFP-21, and MPT-64 antigens for immunodiagnosis (11). Our own previous study identified a cocktail of serodiagnostic antigens for TB, but no single antigen had high sensitivity (18, 19). In the current study, nine antigens (or antigen segments) were combined with non-conserved dominant B-cell epitopes to be a fusion polyprotein and evaluated for TB serodiagnosis to ascertain whether the polyprotein would improve overall sensitivity and specificity compared with individual antigens and other antigen combinations.

## Materials and Methods

### Study Population

Serum samples from healthy individuals and patients with TB were obtained from Shanghai Pulmonary Hospital (Shanghai, China) from August 2016 to December 2017. All patients with TB were diagnosed as newly treated active TB and were determined to require a full course of TB treatment according to TB diagnostic criteria. Diagnostic criteria included sputum culture or smear positivity, typical radiological manifestation and clinical response to anti-TB treatment consistent with active TB. The group with LTBI was recruited from individuals referred to the hospital with suspected TB but displaying no clinical symptoms and subsequent medical evaluation of LTBI based on a positive QuantiFERON-TB Gold (QFT-G; Qiagen) test result. Based on the manufacturer’s information, QFT-G results were interpreted as positive when the value of TB Antigen minus Nil [IU/mL] was > 0.35 IU/mL when only Nil and TB antigen tubes were used, or the value of TB Antigen minus Nil [IU/mL] was > 0.35 IU/mL or Mitogen minus Nil [IU/mL] was > 0.50 IU/mL when Nil, TB antigen, and mitogen tubes were used. The healthy control group included subjects with no history of TB and a negative QFT-G result. Individuals with HIV co-infections, hepatitis infections, other autoimmune disorders, cancer, or chronic diseases were excluded. The study protocol was approved by the ethics committee of Shanghai Pulmonary Hospital, China. Written informed consent was obtained from all subjects before blood sampling. Additional information regarding patients with TB is listed in **Supplementary Table 1**.

### Acquisition of the Lysates From Common Bacteria for Sera Pre-Adsorption

Lysates from Vibrio mimicus, Staphylococcus aureus, Bacillus subtilis, Proteus vulgaris, Staphylococcus epidermidis, Enterobacter aerogenes, γ-Streptococcus, Staphylococcus citreus, and Escherichia coli were used to pre-adsorb the serum samples to block antibodies against bacterial antigens and reduce non-specific reactions. Bacteria were cultured at in a 100-mL volume overnight at 37°C, collected by centrifugation at 13,000 rpm at 4°C for 10 min, resuspended with 10 mL phosphate buffered saline (PBS), and then were subjected to ultrasonic decomposition. The sonicates were centrifuged at 13,000 rpm and the supernatants were collected. Next, 800 µL supernatant, 200 µL sera, and 2 mL PBS were mixed at room temperature for 5 h to adsorb antibodies against bacterial proteins. After centrifugation, the absorbed sera were stored at –20°C until use.

### B-Cell Epitope Selection

ABCpred software (available at https://webs.iiitd.edu.in/raghava/abcpred/ABC_submission.html, Date of access: August 18, 2021) was used online to screen potential B-cell epitopes (20). Key parameters, including probability of surface exposure, local hydrophobicity, beta-turn amino-acid sequence propensity, atomic flexibility and experimental high-performance liquid chromatography (HPLC) retention times of synthetic peptides, were considered in this software. For individual antigens, default parameters, such as threshold (more than 0.80) and overlapping filter, were set.

### BLAST for Non-Conserved Sequences

Protein BLAST from NCBI (available at https://blast.ncbi.nlm.nih.gov/Blast.cgi, Date of access: August 20, 2021) was used to screen each individual antigen to identify sequences that were homologous with those from other bacteria. Firstly, sequences derived from *Mycobacterium* that were highly homologous to the target antigen sequences (greater than 90%) were excluded. Next, sequences derived from three to four types of common bacteria with 50-80% identity and occurring frequently were selected for further analysis. COBALT online web server (available at https://www.ncbi.nlm.nih.gov/tools/cobalt/cobalt.cgi, Date of access: August 20, 2021) was then employed to compare targeted antigen sequences with the above selected sequences, and identify non-conserved sequences (21). Final ready-to- synthesize sequences were obtained by integration of B-cell epitope prediction and BLAST and COBALT results.

### Synthesis of Polypeptides

Polypeptides were synthesized by ChinaPeptides Ltd (Suzhou, China). Purification of recombinant proteins was performed by ion exchange and protein concentrations were determined by the Bradford method (22). Each polypeptide exhibited greater than 95% purity and concentrations of each polypeptide were all =1 mg/mL.

### Three-Dimensional (3D) Structure Prediction of the Fusion Polyprotein

To predict the 3D structure of the fusion polyprotein, I-TASSER online web server (available at https://zhanggroup.org/I-TASSER/, Date of access: August 24, 2021) was used, which is a hierarchical template-based method for protein structure and function (23, 24). For a given polyprotein sequence, I-TASSER firstly identifies super secondary structure motifs from the Protein Data Bank (PDB) library by multiple threading approach LOMETS (25). A higher score indicates a more confident prediction of secondary structure. Normalized B-factor is then predicted and negative values indicates the residue is more stable in the structure. Top 10 threading templates are used by I-TASSER. The alignments are from the top templates, where conserved regions often have higher structure accuracy. Norm. Z-score > 1 indicates a good alignment and the higher, the better. Top five models were further predicted. The confidence of each model is quantitatively measured by C-score that is calculated based on the significance of threading template alignments and the convergence parameters of the structure assembly simulations. C-score is typically in the range of (–2, 5), where a C-score of a higher value signifies a model with a higher confidence and vice-versa. C-score > -1.5 indicates a high-quality structure prediction. TM-score and RMSD are estimated based on C-score and protein length following the correlation observed between these qualities.

Protein disorder was predicted by DEPICTER online web server (available at http://biomine.cs.vcu.edu/servers/DEPICTER/#Help, Date of access: August 24, 2021) (26). Users submits a FASTA-formatted sequence of the input protein using the interface of the server. A subset of predictors, including lupred L, lupred S, and SPOT-Disorder, can be selected to run.

### Polyprotein Expression

Life Technology (Thermo Fisher Scientific, MA, USA) helped to construct the plasma expression vector for cloning and expression of the polyprotein. Codon usage was optimized for *E. coli*. In detail, polyprotein expression in *E. coli Rosetta* (DE3; Novagen, Germany) was induced with 1 mM isopropyl-β-D-thiogalactoside (IPTG) and analyzed by sodium dodecyl sulfate polyacrylamide gel electrophoresis (SDS-PAGE) and Western blotting. The supernatant and inclusion bodies from the expression process were stored for further analysis.

### Indirect ELISA

IgG antibodies were detected by indirect ELISA as previously described (18). Briefly, each purified recombinant antigen or polypeptide was diluted to their optimal concentration with coating buffer (0.05 M Na2CO3-NaHCO3, pH 9.6) and 100 µL was used to coat each well of 96-well Immunosorp plates (Nunc, Denmark) overnight at 4°C. Plates were washed three times with 375 µL PBST (137 mM NaCl, 2.7 mM KCl, 10 mM Na2HPO4, 2 mM KH2PO4, and 0.05% Tween-20), and then blocked in 0.2 mL 5% skimmed milk for 2 h at room temperature. After three washes with 375 μL PBST, test serum samples (diluted 1:50 in blocking buffer) were added and incubated at 37°C for 1 h. After three washes, 100 µL horseradish peroxidase (HRP)-conjugated secondary antibody (1:10,000) (Promega, USA) was added for 0.5 h at 37°C. After five washes, the color reaction was developed by 100 µL TMB (3, 3’, 5, 5’-tetramethylbenzidine) substrate solution (eBioscience, USA) and stopped by the addition of 50 µL 2 N H2SO4 (eBioscience, USA). The optical density (OD) at 450 nm was measured using a microplate reader (ELX50, Bio-Tek Instruments, USA). A novel evolved immunoglobulin-binding molecule (NEIBM)-ELISA method, which was designed to detect human IgG, IgM, and IgA, was also used to detect the antibody level against the polyprotein (27–29).

### Statistical Analysis

Statistical analyses were performed using SPSS (Version 20.0, SPSS Inc., Chicago, IL, USA), GraphPad Prism 8.0 (GraphPad Software Inc., San Diego, CA, USA), and MedCalc for Windows (Version 17.8, Ostend, Belgium) software. All statistical tests were two-sided and *P* < 0.05 was considered statistically significant. Results are expressed as mean ± standard deviation (SD), unless otherwise specified. A positive antibody test was defined as an OD value greater than the cutoff value, i.e., the mean OD value plus three SD from the negative healthy control serum.

## Results

### Using Lysates From Common Bacteria for Sera Pre-Adsorption to Reduce Non-Specific Reactions

**Figure 1:**
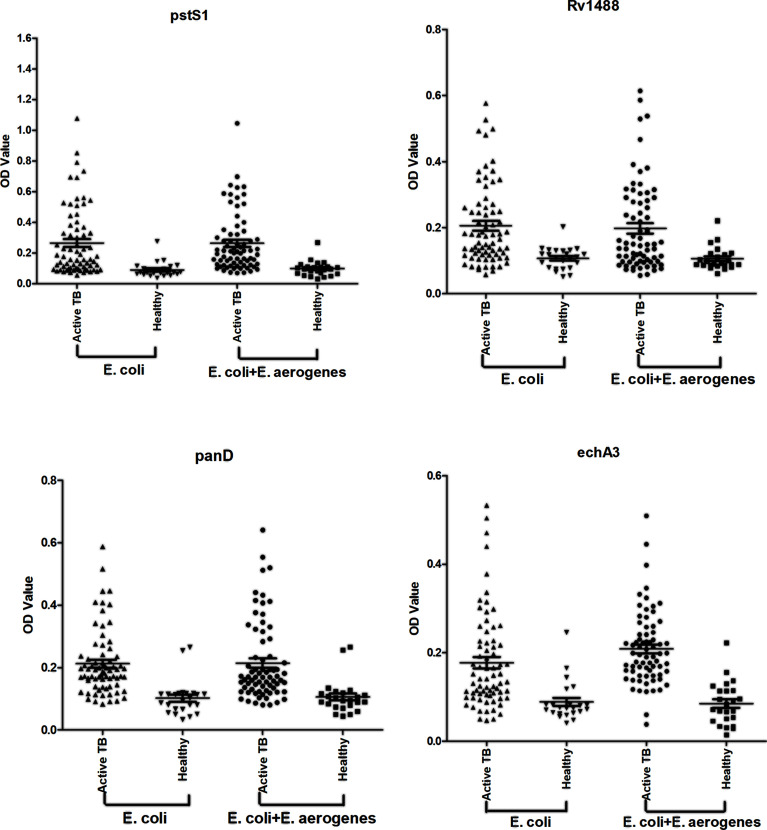
Comparison of the effect of pre-adsorption by *E. coli* and *E. aerogenes* and *E. coli*. A panel of 96 serum samples, including 72 TB patients and 24 healthy controls were recruited. PstS1, Rv1488, PanD and EchA3 four antigens were used to validate it.

Most TB antigens have sequences that are homologous with those of other common bacteria, and TB serum antibodies will inevitably cross-react with antigens from these bacteria. One way to reduce nonspecific reactions is to block antibodies against those bacteria. In this study, three serum samples from healthy individuals, including one strong false positive (SFP), one weak false positive (WFP), and one normal, and one positive serum sample from a patient with TB were used. The serum samples were pre-adsorbed with *Escherichia coli* and other bacterial lysates, including *V. mimicus*, *S. aureus*, *B. subtilis*, *P. vulgaris*, *S. epidermidis*, *Enterobacter aerogenes*, and *S. citreus*, to block antibodies against antigens from these bacteria. There were no significant differences pre-adsorption with *V. mimicus, S. aureus, B. subtilis, S. epidermidis*, and *S. citreus* (**Table 1** and **Supplementary Table 2**). However, the value of serum samples from SFP and WFP markedly decreased after pre-adsorption with *P. vulgaris, Enterobacter aerogenes*, and *Escherichia coli* compared with groups without any bacteria lysates (background groups). Subsequent analyses therefore focused on these three bacteria. Twelve false-positive serum samples from healthy individuals were collected to test pre-adsorption. For protein PstS1, the value of 91.7% (11/12) serum samples significantly decreased after adding *Escherichia coli* lysate, and 88.3% (10/12) decreased after pre-adsorption with *P. vulgaris* or *Enterobacter aerogenes* (**Table 2**). However, for Rv1488, only 75% (8/12) decreased after being pre-adsorbed with *P. vulgaris* (**Supplementary Table 3**). *Escherichia coli* and *Enterobacter aerogen*es were therefore selected for further study. A panel of 96 serum samples comprising 72 patients with TB and 24 healthy controls was employed to compare the difference between *Escherichia coli* and *Escherichia coli & Enterobacter aerogenes*. Four proteins, namely PstS1, Rv1488, PanD and EchA3, were used to validate the panel. There were no significant differences with the addition of *Escherichia coli* and *Enterobacter aerogenes* lysate compared with *Escherichia coli* lysate alone, indicating the redundancy of adding *Enterobacter aerogenes* lysate (**Figure 1** and **Table 3**). However, despite partially increasing the specificity of the serodiagnosis assay, false-positive ratio remained very high for the healthy controls, which warranted exploration of different measures to solve this problem.

**Table 1:** The profile of using lysates from various bacteria for sera pre-adsorption to reduce non-specific reactions (for pstS1).

pstS1	*V. mimicus*	*S. aureus*	*B. subtilis*	*S. epidermidis*	*P. vulgaris*	*E. aerogenes*	*γ-streptococcus*	*S. citreus*	*E. coli*	Mixed	BG	NC
**SFP**	0.563	0.531	0.592	0.543	0.333	0.399	0.512	0.515	0.318	0.405	0.582	0.016
**WFP**	0.325	0.283	0.289	0.274	0.133	0.109	0.247	0.223	0.190	0.156	0.271	0.009
**SP**	0.776	0.784	0.783	0.772	0.618	0.602	0.712	0.678	0.611	0.681	0.698	0.021
**HC**	0.134	0.139	0.122	0.117	0.088	0.085	0.118	0.115	0.064	0.055	0.114	0.017

BG, Background; SFP, Strong false positive; WFP, Weak false positive; SP, strong positive; NC, Negative control; V. mimicus, Vibrio mimicus; S. aureus, Staphylococcus aureus; B. subtilis, Bacillus subtilis; P. vulgaris, Proteus vulgaris; S.epidermis, Staphylococcus epidermidis; E. aerogenes, Enterobacter aerogenes; γ-Streptococcus, gamma-streptococcus; S. citreus, Staphylococcus citreus; E. coli, Escherichia coli. The measuring value is OD.

**Table 2:** Comparison of the effect of pre-adsorption by *P. vulgaris, E. aerogenes* and *E. coli (*for *pstS1)*.

pstS1	BG	*E. coli*	*E. aerogenes*	*P. vulgaris*	Mixed	Blank
**FP1**	0.625	0.246	**0.565**	**0.565**	0.267	0.024
**FP2**	0.541	0.288	0.331	0.294	0.236	0.011
**FP3**	0.403	0.268	0.297	0.291	0.269	0.01
**FP4**	0.23	0.201	0.187	0.176	0.186	0.01
**FP5**	0.373	**0.34**	**0.336**	**0.347**	0.347	0.011
**FP6**	0.262	0.174	0.195	0.18	0.177	0.01
**FP7**	0.946	0.415	0.468	0.493	0.52	0.017
**FP8**	0.283	0.13	0.181	0.166	0.144	0.011
**FP9**	1.007	0.246	0.326	0.336	0.51	0.011
**FP10**	0.542	0.17	0.247	0.188	0.218	0.009
**FP11**	0.513	0.167	0.225	0.218	0.212	0.01
**FP12**	0.532	0.181	0.205	0.246	0.259	0.011
**SP1**	0.504	0.316	0.271	0.263	0.253	0.009
**SP2**	0.455	0.204	0.259	0.329	0.252	0.009
**HC1**	0.121	0.086	0.135	0.125	0.087	0.016
**HC2**	0.109	0.054	0.062	0.063	0.087	0.01

E. aerogenes, Enterobacter aerogenes; E. coli, Escherichia coli; BG, Background; FP, False positive; SP, strong positive; HC, Healthy control. The measuring value is OD. Bold values indicated that the value of corresponding serum samples did not significantly decrease after adding bacteria lysates.

**Table 3:** Comparison of the effect of pre-adsorption by *E. coli* and *E. aerogenes* & *E. coli*.

Rv.	*Vs*	True positive	False negative	True negative	False positive	Sensitivity (%, 95% CI)	Specificity (%, 95% CI)	P Value
pstS1	*E. coli*	29	43	23	1	40.3 (28.9–52.5)	95.8 (78.9–99.9)	P1 = 0.86
	*E. coli+E. aerogenes*	27	45	23	1	37.5 (26.4–49.7)	95.8 (78.9–99.9)	P2 = 1.00
Rv1488	*E. coli*	27	45	23	1	37.5 (26.4–49.7)	95.8 (78.9–99.9)	P1 = 1.00
	*E. coli+E. aerogenes*	26	46	23	1	36.1 (25.1–48.3)	95.8 (78.9–99.9)	P2 = 1.00
PanD	*E. coli*	17	55	22	2	23.6 (14.4–35.1)	91.7 (73.0–99.0)	P1 = 1.00
	*E. coli+E. aerogenes*	17	55	22	2	23.6 (14.4–35.1)	91.7 (73.0–99.0)	P2 = 1.00
EchA3	*E. coli*	19	53	23	1	26.4 (16.7–38.1)	95.8 (78.9–99.9)	P1 = 1.00
	*E. coli+E. aerogenes*	20	52	23	1	27.8 (17.9–39.6)	95.8 (78.9–99.9)	P2 = 1.00

E. aerogenes, Enterobacter aerogenes; E. coli, Escherichia coli; Data was analyzed by McNemar’s test by MedCalc. P1, P value of the sensitivity and P2, P value of the specificity.

### Synthesizing Polypeptides to Reduce Non-Specific Reactions

**Figure 2:**
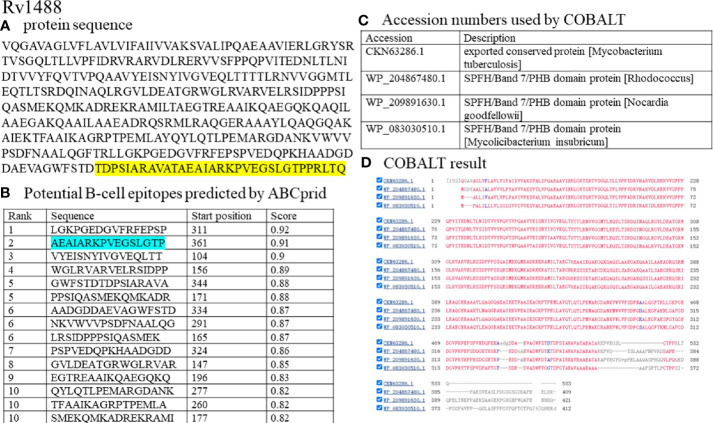
Synthesizing the polypeptide of Rv1488 antigen with dominant B-cell epitopes and non-conserved fragments. **(A)** the full protein sequence of Rv1488 antigen. Sequences colored in yellow represented the polypeptide of Rv1488 Synthesized. **(B)** ABCprid software was used to screen potential B-cell epitopes. Residues colored in green represented dominant B-cell epitopes predicted. **(C)** The accession numbers of bacteria used for COBALT software analysis; **(D)** The result of COBALT software analysis. This view showed residue conservation: red for conserved residues, blue for columns with no gaps, and gray for columns containing gaps.

Another measure to reduce non-specific reactions is to synthesize polypeptides that contained dominant B-cell epitopes and non-conserved fragments compared with other common bacteria. ABCpred software was used to screen potential B-cell epitopes and COBALT software was further employed to compare and select non-conserved segments of antigens. For example, the full sequence of Rv1488 was submitted to ABCpred software setting the parameters as Threshold (0.80) and overlapping filter (**Figure 2A**). As shown in **Figure 2B**, 15 potential B-cell epitopes were predicted. Residues colored in green represented dominant B-cell epitopes predicted. Simultaneously, BLAST and COBALT from NCBI were employed to screen and identify homologous sequences of Rv1488 antigen compared with other common bacteria, such as *Escherichia coli*, and non-conserved segments were selected (**Figure 2C**). The sequence “AADGDDAEVAGWFSTDTDPSIARAVATAEAIARKPVEGSLGTPPRLTQ” was non-conserved, and contained a dominant B-cell epitope “AEATARKPVEGSLGTP” (**Figure 2D**). Thus, the final ready-to- synthesize sequence (Pep-Rv1488), i.e. “TDPSIARAVATAEAIARKPVEGSLGTPPRLTQ” was obtained by integration of the B-cell epitope prediction and non-conserved segments BLAST results. Similar results were obtained for PstS1 and six other antigens (**Supplementary Figure 1**).

To test the effect of pre-adsorption by *Escherichia coli* on the peptides, eight false-positive serum samples from different healthy individuals were collected for each peptide. Two serum samples from normal healthy individuals and two positive serum samples from patients with TB were used as controls. The value of most false-positive serum samples significantly decreased after addition of *Escherichia coli* lysate (**Supplementary Table 4**). The efficiency of reducing non-specific reactions ranged from 62.5% (5/8) to 87.5% (7/8), which demonstrated the effect of pre-adsorption by *Escherichia coli.*

**Figure 3:**
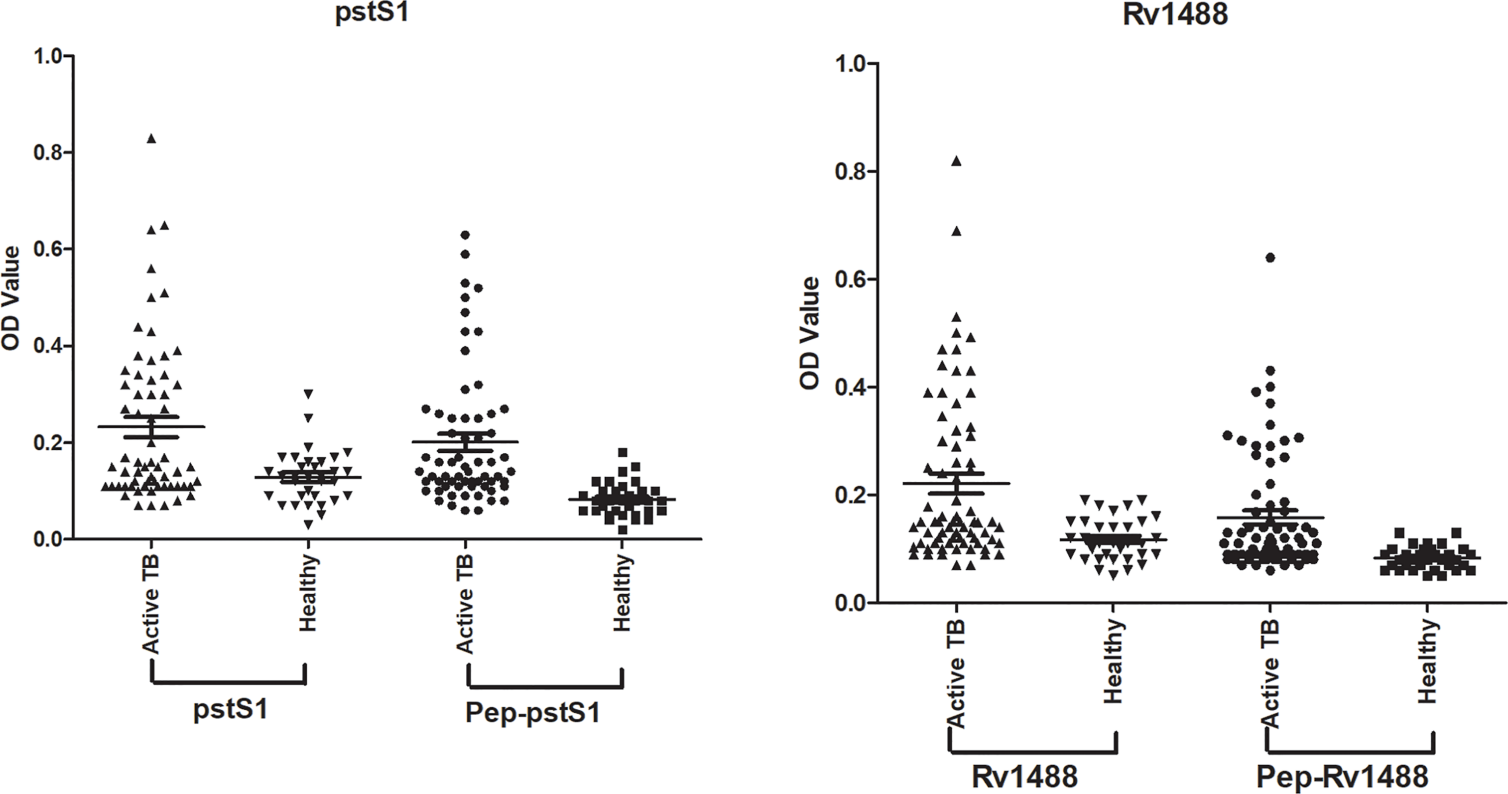
Comparison of the differences of sensitivities and specificities between polypeptides and their corresponding antigens. A panel of 96 serum samples, including 63 TB patients and 33 healthy individuals, was collected to compare the differences between Pep-PstS1 and Pep-Rv1488 and their corresponding antigens.

To assess the potential diagnostic value of the polypeptides, a panel of 96 serum samples comprising 63 patients with TB and 33 healthy individuals, was collected to compare the differences in sensitivities and specificities between these polypeptides and their corresponding antigens. The levels of antibodies against Pep-PstS1 and Pep-Rv1488 in patients with TB and healthy individual groups were significantly decreased when compared with their corresponding antigens (**Figure 3**), which may be due to the loss of some other B-cell epitopes. However, the sensitivities of each polypeptide slightly decreased (P = 0.38 and P = 0.69) (**Table 4**). Conversely, some false-positive serum samples showed seroconversion when tested by polypeptides, which improved their specificities (**Figure 3**). These observations indicated that identification of suitable polypeptides would help effectively reduce non-specific reactions without markedly decreasing sensitivities.

**Table 4:** Comparison of the differences of sensitivities and specificities between polypeptides and their corresponding antigens.

Rv.	True positive	False negative	True negative	False positive	Sensitivity (%, 95% CI)	Specificity (%, 95% CI)	P Value
pstS1	25	38	32	1	39.7 (27.6–52.8)	97.0 (84.2–99.9)	P1 = 0.38; P2 = 1.00
Pep-pstS1	22	41	33	0	34.9 (23.3–48.0)	100.0 (89.4–100.0)	
Rv1488	20	43	32	1	31.8 (20.6–44.7)	97.0 (84.2–99.9)	P1 = 0.69; P2 = 1.00
Pep-Rv1488	18	45	33	0	28.6 (17.9–41.4)	100.0 (89.4–100.0)	

Data was analyzed by McNemar’s test by MedCalc. P1, P value of the sensitivity; P2, P value of the specificity.

### Assembly of the Fusion Polyprotein

In the search for appropriate diagnostic antigens for TB, it was already recognized that no single antigen-based assay had achieved an optimal serodiagnostic performance to date due to the complexity of the human immune response to TB antigens (30). Thus, strategies using multiple antigens either individually or as fusion polyproteins (or segments) have been recommended. However, when the antigens were mixed and tested as one for each patient, sensitivity decreased (**Supplementary Figure 2**). Thus, a novel *M. tb* fusion polyprotein containing multiple polypeptides was preferentially constructed and expressed as an antigen with multi-epitopes.

**Figure 4:**
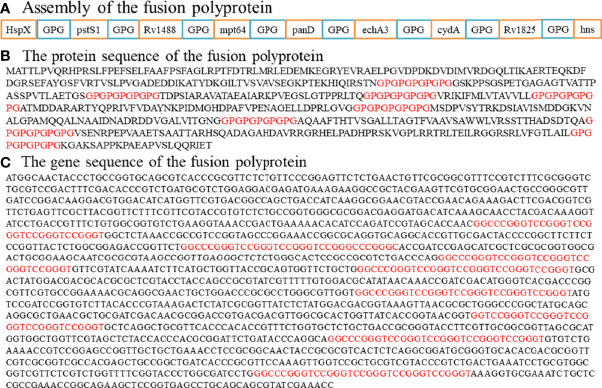
Assembly of the fusion polyprotein. **(A)** HspX and other eight antigen segments were fused as an entirety by a sequence linker (GPGPGPGPGP). **(B)** The protein sequence of the fusion polyprotein. **(C)** The gene sequence of the fusion polyprotein.

Nine antigens, previously identified as exhibiting TB serodiagnostic potential, would be fused as a polyprotein (**Supplementary Table 5**). Since HspX was a serodiagnostic antigen with 33.33% sensitivity and 100% specificity (18), and expression of the fusion molecule HspX with other antigens increased by about 50% as compared with those of the individual antigen, resulting in cheaper production of the fusion antigens (31), the whole sequence of HspX was retained in the fusion protein to enhance immunogenicity. For the other eight antigens, polypeptides were screened as described in the Materials and Methods and fused as an entirety by glycine- and proline-rich (GPGPGPGPGPG) spacers. Finally, a fusion polyprotein containing nine antigens or segments was constructed (**Figure 4**). The polyprotein sequence and its underlying expression-vector/gene sequence have been deposited at NCBI (GenBank accession number MZ956586). Codon usage of the polyprotein was adapted to the codon bias of *Escherichia coli* genes (**Supplementary Figure 3**), and regions of very high (>80%) or very low (<30%) GC content were avoided where possible. During the optimization process the following cis-acting sequence motifs were avoided where applicable: internal TATA-boxes, chi-sites, and ribosomal entry sites; AT-rich or GC-rich sequence stretches; RNA instability motifs; repeat sequences and RNA secondary structures; (cryptic) splice donor and acceptor sites in higher eukaryotes. Final successful optimization involved elimination of negative cis-acting sites (such as splice sites, TATA-boxes, etc.), which may negatively influence expression, wherever possible. GC content was adjusted to prolong mRNA half-life. Codon usage adapted to the bias of *Escherichia coli* resulted in a CAI* value of 0.95. The optimized gene should therefore allow high and stable expression rates in *Escherichia coli*.

### 3D Structure Prediction of the Fusion Polyprotein

The use of homology modeling (HM) was always available for 3D structure prediction of most proteins (32). However, it was questionable in the current study as the fusion polyprotein was artificially constructed with the “tail” sequence added to HspX consisting of diverse protein segments linked by glycine- and proline-rich (GPGPGPGPGPG) spacers, such that HM may be unable to reliably predict the polyprotein structure, and whatever structure had thus been obtained by HM was potentially misleading. Therefore, it might be more suitable using I-TASSER software, which combined homology modeling and *de novo* (ab initio) prediction strategies. Prediction of the secondary structure suggested that the polyprotein was an alpha-beta protein, which contains 17 alpha-helices (in red) and 17 beta-strands (in blue) (**Supplementary Figure 4A**). The predicted solvent accessibility was presented in 10 levels, from buried (0) to highly exposed (9) (**Supplementary Figure 4B**). The normalized B-factor was predicted and the regions at the N- and C-terminals and most of the loop regions were predicted with positive values, indicating that these regions enriched for linear/continuous B-cell epitopes were structurally more flexible and disordered than other regions (**Supplementary Figure 4C**). Similar observation was made using DEPICTER for the prediction of protein disorders of the polyprotein (**Supplementary Figure 5**). Top 10 threading templates were used and top five models were further predicted with global and local accuracy estimations (**Supplementary Figures 4D, E**). The C-score of the first model was -1.79, with an estimated TM-score = 0.50 ± 0.15 and RMSD = 11.8 ± 4.5Å relative to the native. The prediction with low C-score value indicated the lack of good templates in the protein structure library, and ab initio modeling of medium-to-large size proteins without using templates remained to be improved.

### Assessment of the Sensitivity and Specificity of the Fusion Polyprotein

The optimized gene was amplified by PCR using the primers F-*E.coli* 5’-CGGATCCGGCTCTAAACCGCCGTCCG-3’ and R-*E.coli* 5’-CCTCGAGGGTTTCGATACGCTGCTGCAG3-’. The constructed plasma expression vector was cloned and expressed in *E. coli* Rosetta and analyzed by SDS-PAGE and Western blotting. The purity of the fusion polyprotein was 90.8%, as determined by Quantity One software (**Supplementary Figure 6**).

To test the effect of pre-adsorption by *Escherichia coli* for the polyprotein, eight false-positive serum samples from different healthy individuals were used. The value of 87.5% (7/8) serum samples significantly decreased after adding *Escherichia coli* lysate (**Supplementary Table 5**), which was consistent with previous results.

**Figure 5:**
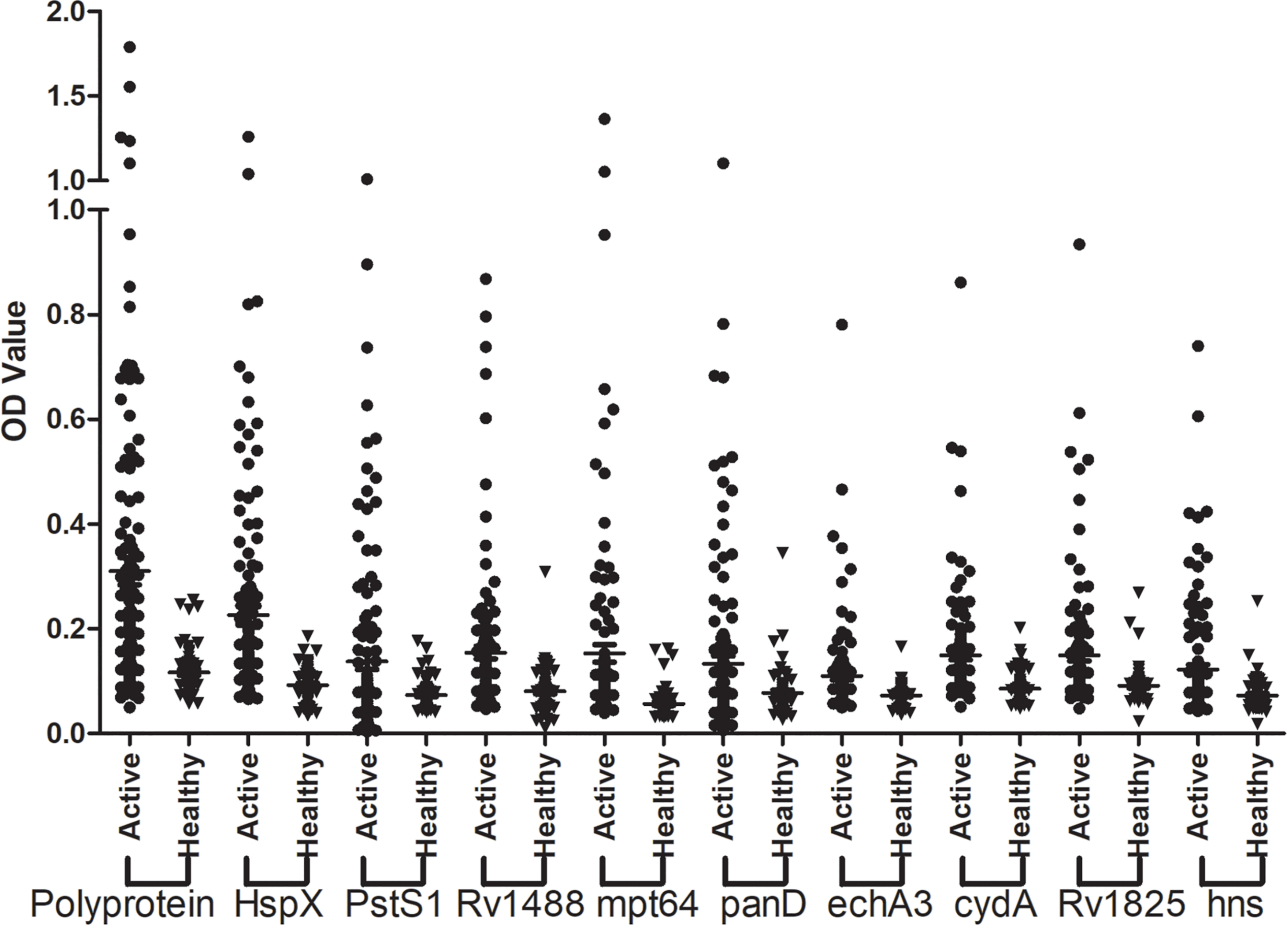
Assessment of the sensitivity and specificity of the fusion polyprotein. The indirect ELISA assay was conducted and the sensitivity and specificity of the polyprotein and each individual antigen or segments was evaluated.

The sensitivity and specificity of the polyprotein was evaluated using an indirect ELISA and a panel of 192 serum samples comprising 128 from patients with TB and 64 from healthy individuals (**Figure 5** and **Supplementary Table 6**). The sensitivity of the polyprotein was 60.2%, which was higher than that of HspX and other individual antigen segments. The specificity of the polyprotein was 93.8%, which was not significantly decreased (**Table 5**). To further evaluate the factors affecting diagnosis of the polyprotein, patients with TB were divided into different subpopulations based on clinical backgrounds. The sensitivities of the polyprotein in sputum smear-positive and -negative samples were similar, with no significant difference (63.3% *vs* 58.2%, P = 0.57). Similar observations were made between chest X-ray-positive and -negative samples (55.6% *vs* 62.0%, P = 0.51) (**Supplementary Table 7**). To evaluate whether the detection of whole antibodies would improve the sensitivities of the polyprotein, a panel of 48 serum samples from patients with TB and 16 serum samples from healthy individuals were independently collected and horseradish peroxidase (HRP)-labeled LD5 (HRP-LD5) was used to detect human IgG, IgM, and IgA. The sensitivities of the polyprotein for IgG and LD5 detection were 64.6% and 52.1%, respectively, with no significant difference (P = 0.29), and the specificities were both 93.8%, with no significant difference (P = 1.00) (**Supplementary Table 8**).

**Table 5:** Sensitivities and specificities of nine individual antigens (or segments) and the fusion polyprotein with sera from TB patients and healthy controls.

Rv.	Sensitivity (%, 95% CI)	Specificity (%, 95% CI)
HspX	31.3 (23.4–40.0)	100 (94.4–100.0)
pstS1	25.0 (17.8–33.4)	98.4 (91.6–100.0)
Rv1488	23.4 (16.4–31.7)	98.4 (91.6–100.0)
mpt64	18.0 (11.7–25.7)	96.9 (89.2–99.6)
panD	18.0 (11.7–25.7)	96.9 (89.2–99.6)
echA3	12.5 (7.3–19.5)	98.4 (91.6–100.0)
cydA	20.3 (13.7–28.3)	98.4 (91.6–100.0)
Rv1825	15.6 (9.8–23.1)	95.3 (86.9–99.0)
hns	18.0 (11.7–25.7)	98.4 (91.6–100.0)
Polyprotein	60.2 (51.1–68.7)	93.8 (84.3–98.2)

CI, confidence intervals.

## Discussion

Serology-based tests for TB diagnosis, though rapid, efficient and easily implemented, have exhibited unsatisfactory and suboptimal levels of sensitivity and specificity to date. One possible reason for this is the heterogeneity of the antibody response in patients with TB. The number and types of seropositive antigens vary from person to person and this variation may be linked to genetic polymorphisms of the human leukocyte antigen (HLA) class II alleles (30). WHO does not recommend the use of the current commercial serological tests for TB diagnosis but does still encourage further research and development in this field.

Due to TB antigens having sequences homologous with those of BCG and other common bacteria, TB serum antibodies would inevitably cross-react with antigens from these bacteria, thus inducing high false-positive rates and reducing the specificity of the serodiagnostic tests. Blocking antibodies against these bacteria is a feasible way to reduce nonspecific reactions. In the current study, in addition to *Escherichia coli* that had been used previously, lysates from other bacteria, including *V. mimicus, S. aureus, B. subtilis, P. vulgaris, S. epidermidis, Enterobacter aerogenes*, and *S. citreus*, were used to pre-adsorb the serum samples to block antibodies against bacterial antigens. There were no significant differences from adding *V. mimicus, S. aureus, B. subtilis, S. epidermidis*, and *S. citreus*, and the value of serum samples from strong false-positive and weak false-positive groups significantly decreased after pre-adsorption with *P. vulgaris, Enterobacter aerogenes*, and *Escherichia coli* compared with the groups without any bacterial lysate. Further study analysis revealed that *Escherichia coli* and *Enterobacter aerogen*es performed better. There were no significant differences resulting from the addition of a combined *Escherichia coli* and *Enterobacter aerogenes* lysate compared with only *Escherichia coli*, indicating the redundancy of adding *E. aerogenes* lysate. However, despite partially increasing the specificities, there was still a large false-positive ratio for healthy controls, hence, exploration of further measures was required to solve this problem.

Synthesizing peptides according to immunodominant antigens of *M. tb* could be an alternative and effective approach for serodiagnosis. B-cell epitope-containing peptides of RD1 (ESAT-6, CFP-10) and RD2 (CFP-21, MPT-64) antigens were used for immunodiagnosis of pulmonary TB (11). Afzal et al. constructed fusion proteins tn1FbpC1-tnPstS1 and tn2FbpC1-tnPstS1 with immunodominant B-cell epitope sequences and found that removal of a non-epitopic FbpC1 region (amino-acid residues 34–96) unmasked some of the epitopes, resulting in greater sensitivity (12).In the current study, these peptides should contain non-conserved fragments of TB antigens compared with other bacteria, as determined by bioinformatics analysis, and simultaneously retain dominant B-cell epitopes where possible. To investigate whether pre-adsorption with *Escherichia coli* lysate reduced non-specific reactions of these peptides, eight false-positive serum samples from different healthy individuals were collected and assayed. The value of most false-positive serum samples significantly decreased, with the efficiency of reducing non-specific reactions ranging from 62.5% (5/8) to 87.5% (7/8). Moreover, a panel of 96 serum samples, comprising 63 patients with TB and 33 healthy individuals, was used to compare the potential diagnostic value of these polypeptides with their corresponding antigens. Although the levels of antibodies against these polypeptides in groups of patients with TB and healthy individuals significantly decreased, the sensitivities of each polypeptide only slightly decreased (P = 0.38 and P = 0.69) while their specificities improved. These findings indicated that identification of suitable proper polypeptides would facilitate effective reduction of non-specific reactions without markedly decreasing sensitivities.

Since it was impossible to achieve optimal serodiagnostic performance by using a single antigen-based assay, strategies using multiple antigens either individually or as fusion polyproteins (or segments) have been recommended. Our preliminary experiment revealed that a crude mixture of several antigens did not significantly increase the rates of positive reactions, and was even counterproductive (**Figure S2**). A strategy of constructing and expressing a fusion polyprotein containing multiple polypeptides was therefore explored. Our previous study identified a cocktail of serodiagnostic antigens for active TB (18) and the segments from nine of these antigens were combined with non-conserved dominant B-cell epitopes as a fusion polyprotein in the current study, which may improve overall sensitivity and specificity compared with individual antigen and other combination forms. Since HspX was a serodiagnostic antigen with 33.33% sensitivity and 100% specificity in our previous study (18), and expression of the fusion molecule HspX with other antigens was increased by about 50% as compared to those of the individual antigen, resulting in cheaper production of the fusion antigens (32), the whole sequence of HspX was retained in the fusion protein to enhance immunogenicity. Of the other eight antigens, pstS1 and mpt64 were the most frequently studied, while Rv1488, panD, echA3, cydA, Rv1825 and hns were novel antigens identified in our previous study. The 3D theoretical structure of the polyprotein comprising nine antigens or segments was predicted and the codon bias was adapted for *Escherichia coli* genes. In the current study, it was unreasonable to use HM models to predict the 3D structure of the polyprotein since it was artificially constructed, such that structure obtained by HM was potentially misleading and unrealistic. A combination of ab initio protein structure prediction and protein disorder prediction (using I-TASSER and DEPICTER) was used to replace HM models (32). The normalized B-factor prediction indicated that the regions of the polyprotein enriched for linear/continuous B-cell epitopes were structurally more flexible and disordered. Similar observation was made using DEPICTER. However, the 3D model prediction with low C-score value indicated this artificially constructed polyprotein lacked good templates in the protein structure library, and ab initio modeling of medium-to-large size proteins without using templates remained to be improved. As Roy et al. suggested (23), other sources of structural information, such as data from mutagenesis or crosslinking experiments on the target protein, can be specified as external restraints to improve the modeling quality.

The fusion polyprotein was successfully expressed and an indirect ELISA assay was conducted to evaluate the sensitivity and specificity of the polyprotein. The sensitivity of the polyprotein was 60.2%, which was higher than that of HspX and other individual antigen segments. The specificity of the polyprotein was 93.8%, which was not significantly decreased compared with HspX and other individual antigen segments. Previous studies indicated that there were some associations between clinical backgrounds, including sex, age, bacterial loads, and chest X-ray status, and antibody reactivity of TB diagnostic antigens (33). However, there were no significant differences between the sensitivities of the polyprotein in sputum smear-positive and -negative samples (63.3% *vs* 58.2%, P = 0.57), and in chest X-ray-positive and -negative samples (55.6% *vs* 62.0%, P = 0.51) in the current study. IgM and IgA detection can supplement IgG detection in advanced TB and the simultaneous detection of IgG/IgM/IgA may improve the positivity rate. Li et al. found that the mixture of anti-human IgG and IgM added to a well [Ig(G + M)] had a stronger immunoreactivity to PstS1-LEP than the single antibody (34). Abebe et al. found that there were significant variations in IgA, IgG, and IgM responses to the different antigens, but not all antibody isotype responses are markers of clinical TB (35). Here we used HRP-LD5 to detect human IgG, IgM, and IgA. However, the sensitivities and specificities of the polyprotein for IgG and LD5 detection were both not significant difference. The lack of unified standards for antibody-based diagnosis made different studies controversial and variable, implying further research and development in this field.

In summary, serological tests represent an attractive option for TB diagnosis. However, the unsatisfactory sensitivities and specificities of the currently available options based on single or multiple target antigens do not yet meet the requirements for clinical use. This study therefore explored measures to solve this problem. Lysates from common bacteria to block antibodies against these bacteria were used in serum samples and non-conserved dominant B-cell epitopes of antigens were synthesized to reduce nonspecific reactions and thus improve the specificity of the serological tests. Furthermore, construction and evaluation of a fusion polyprotein containing HspX and eight other antigen segments revealed that the sensitivity of the polyprotein was 60.2%, which was higher than that of HspX and other individual antigen segments, while there was no significant decrease in the specificity of the polyprotein (93.8%). This study demonstrates the roles of fusion polyproteins in the humoral immune response against TB infection and provides a potential novel approach for development of TB diagnostics.

## Data Availability Statement

The raw data supporting the conclusions of this article will be made available by the authors, without undue reservation.

## Ethics Statement

The studies involving human participants were reviewed and approved by Naval Medical University. The patients/participants provided their written informed consent to participate in this study.

## Author Contributions

FZ and WP conceived and designed the experiments. FZ, XX, and XC performed the experiments. FZ analyzed the data. FZ collected the samples. FZ and WP drafted the manuscript. All authors reviewed the manuscript. All authors contributed to the article and approved the submitted version.

## Funding

This work was supported by Foundation of Shanghai Municipal Commission of Health and Family Planning (GWV-10.2-YQ05), Shenzhen Science Founding (JCYJ20170307095037263), Chinese Postdoctoral Science Foundation (2017M612849) and National S & T Program (2013ZX10003007) in China.

## Conflict of Interest

The authors declare that the research was conducted in the absence of any commercial or financial relationships that could be construed as a potential conflict of interest.

## Publisher’s Note

All claims expressed in this article are solely those of the authors and do not necessarily represent those of their affiliated organizations, or those of the publisher, the editors and the reviewers. Any product that may be evaluated in this article, or claim that may be made by its manufacturer, is not guaranteed or endorsed by the publisher.

## Supplementary Material

Supplementary Figure 1 Synthesizing the polypeptide of PstS1 and six other antigens with dominant B-cell epitopes and non-conserved fragments. **(A)**. the full protein sequence of PstS1 or six other antigens. Sequences colored in yellow represented the polypeptide of PstS1 or six other antigens Synthesized. **(B)**. ABCprid software was used to screen potential B-cell epitopes. Residues colored in green represented dominant B-cell epitopes predicted. **(C)**. The accession numbers of bacteria used for COBALT software analysis; **(D)**. The result of COBALT software analysis. This view showed residue conservation: red for conserved residues, blue for columns with no gaps, and gray for columns containing gaps.

Supplementary Figure 2 Combination of multiple antigens for serodiagnosis. Different coating forms were assessed to test the sensitivities and specificities.

Supplementary Figure 3 Adaption of the codon usage of the polyprotein for the codon bias of *E. coli* genes. Codon quality distribution, codon quality plot and GC content were analyzed.

Supplementary Figure 4 The predicted 3D structure of the fused polyprotein. **(A)**. The secondary structure was predicted. “H,” “S,” and “C” indicated helix, strand, and coil, respectively. **(B)**. The predicted solvent accessibility was presented in 10 levels, from buried (0) to highly exposed (9). **(C)**. The normalized B-factor was predicted. Positive values indicated the residue is less stable in the structure. **(D)**. Top 10 threading templates are used by I-TASSER. The alignments are from the top templates, where conserved regions often have higher structure accuracy. Norm. Z-score > 1 indicates a good alignment and the higher, the better. **(E)**. Top five models were predicted. C-score > -1.5 indicates a high-quality structure prediction.

Supplementary Figure 5 Protein disorder of the polyprotein was predicted by DEPICTER. Disordered Residue Predictors, including lupred L, lupred S, and SPOT-Disorder, were used.

Supplementary Figure 6 The purification of the fusion polyprotein. Purified polyprotein was examined by SDS-PAGE and quantified using the Beyotime BCA protein quantitation kits. The purity was determined by Quality One software.
