# Supplementary material for: Development and Evaluation of a Fusion Polyprotein Based on HspX and Other Antigen Sequences for the Serodiagnosis of Tuberculosis
Source: Front Immunol. 2021 Oct 4;12:726920. doi: 10.3389/fimmu.2021.726920 (PMC8521024; doi:10.3389/fimmu.2021.726920)
Supplement: Supplementary Figure 1 — Synthesizing the polypeptide of PstS1 and six other antigens with dominant B-cell epitopes and non-conserved fragments. (A). the full protein sequence of PstS1 or six other antigens. Sequences colored in yellow represented the polypeptide of PstS1 or six other antigens Synthesized. (B). ABCprid software was used to screen potential B-cell epitopes. Residues colored in green represented dominant B-cell epitopes predicted. (C). The accession numbers of bacteria used for COBALT software analysis; (D). The result of COBALT software analysis. This view showed residue conservation: red for conserved residues, blue for columns with no gaps, and gray for columns containing gaps. [file DataSheet_1.docx]

**Supplementary Figure 1**


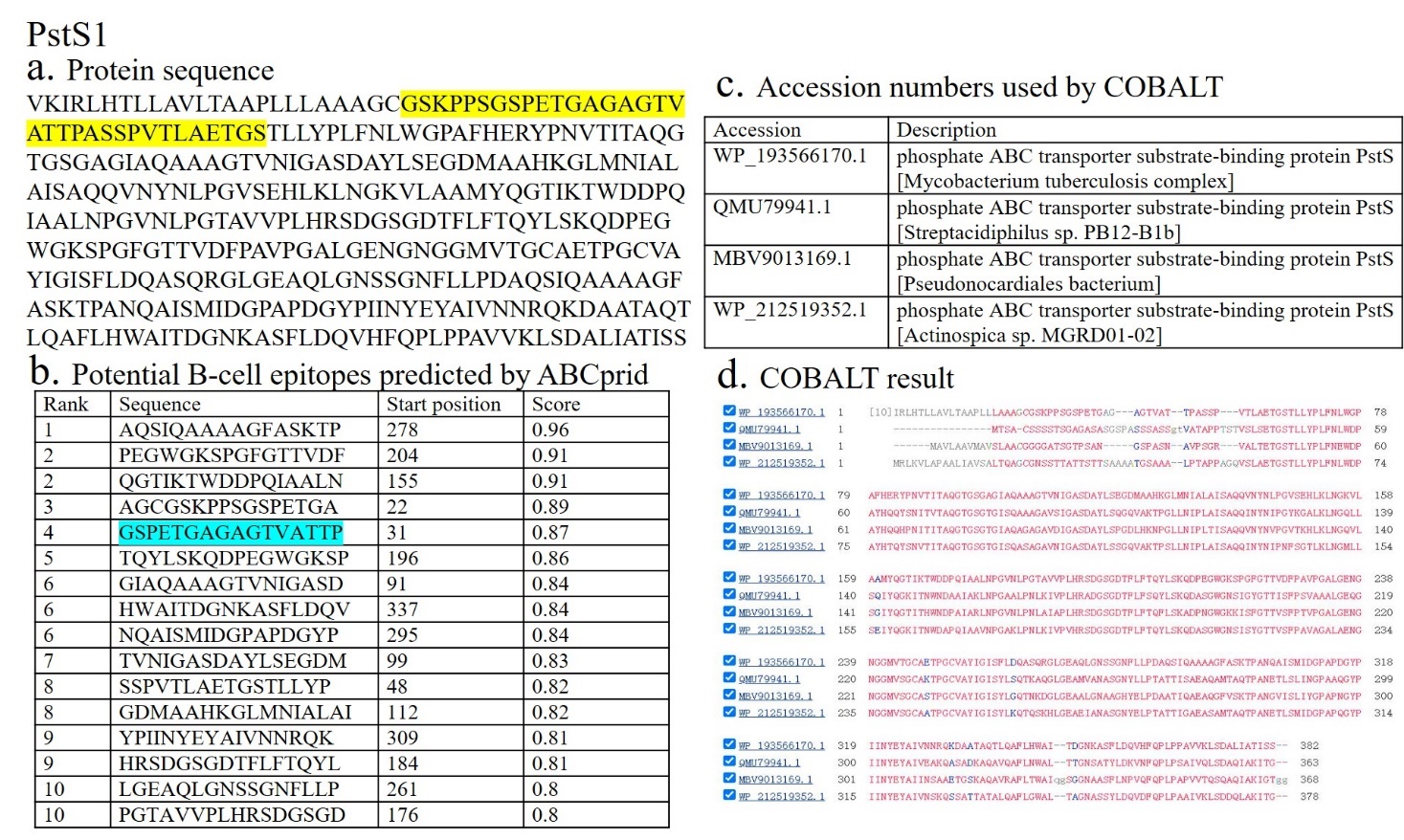


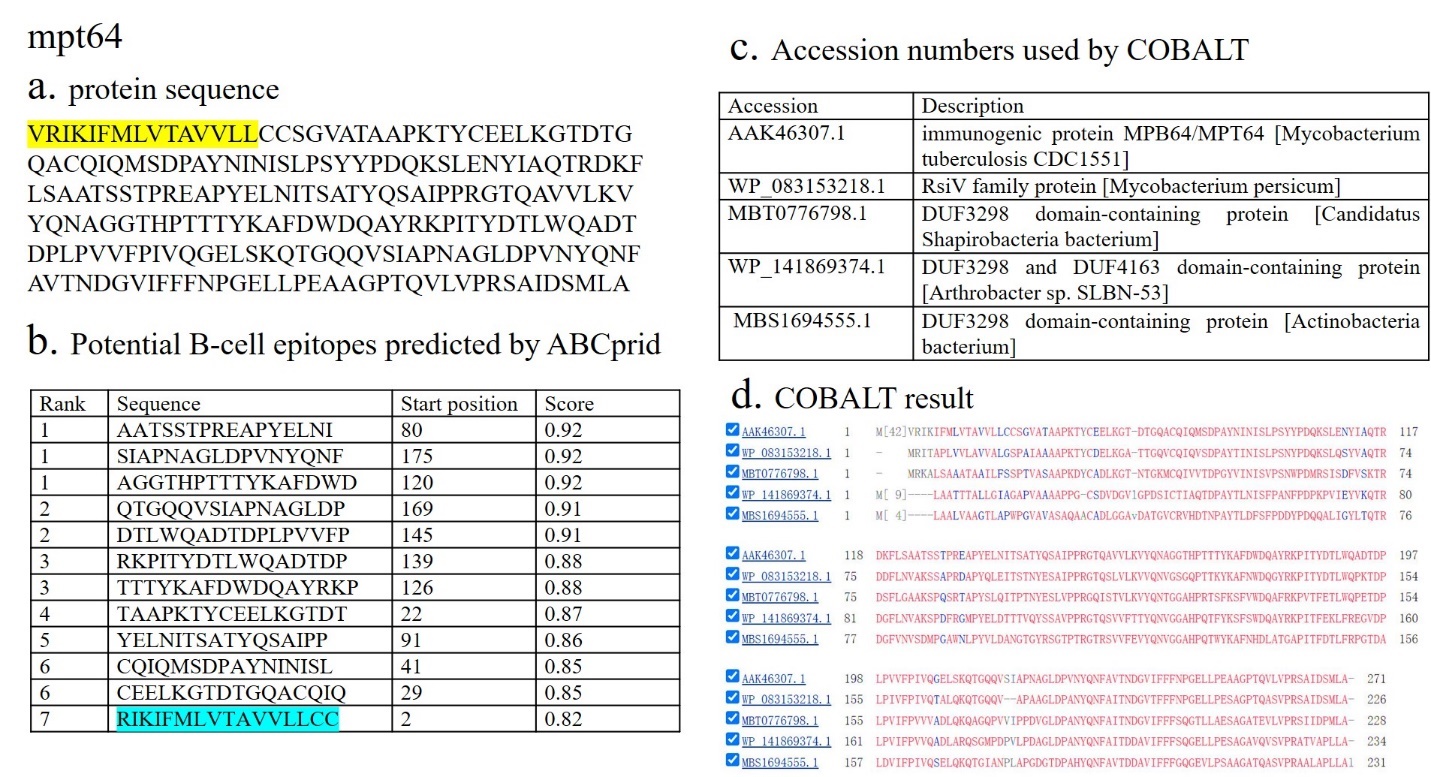


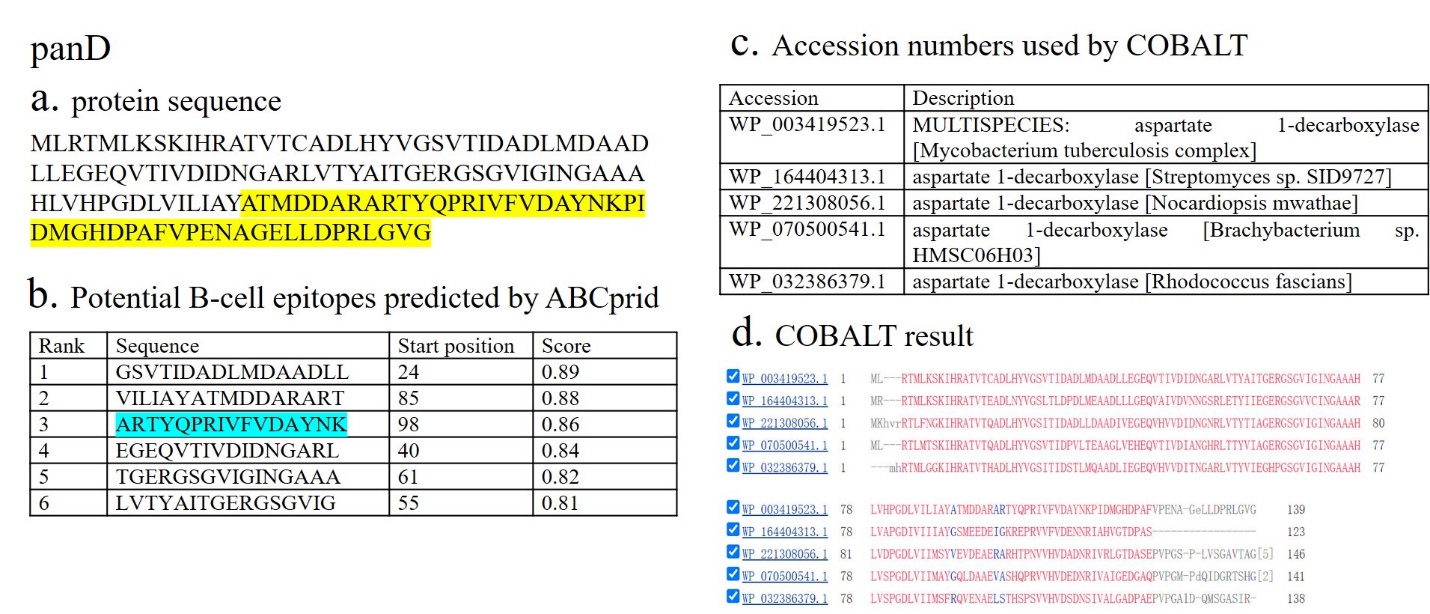


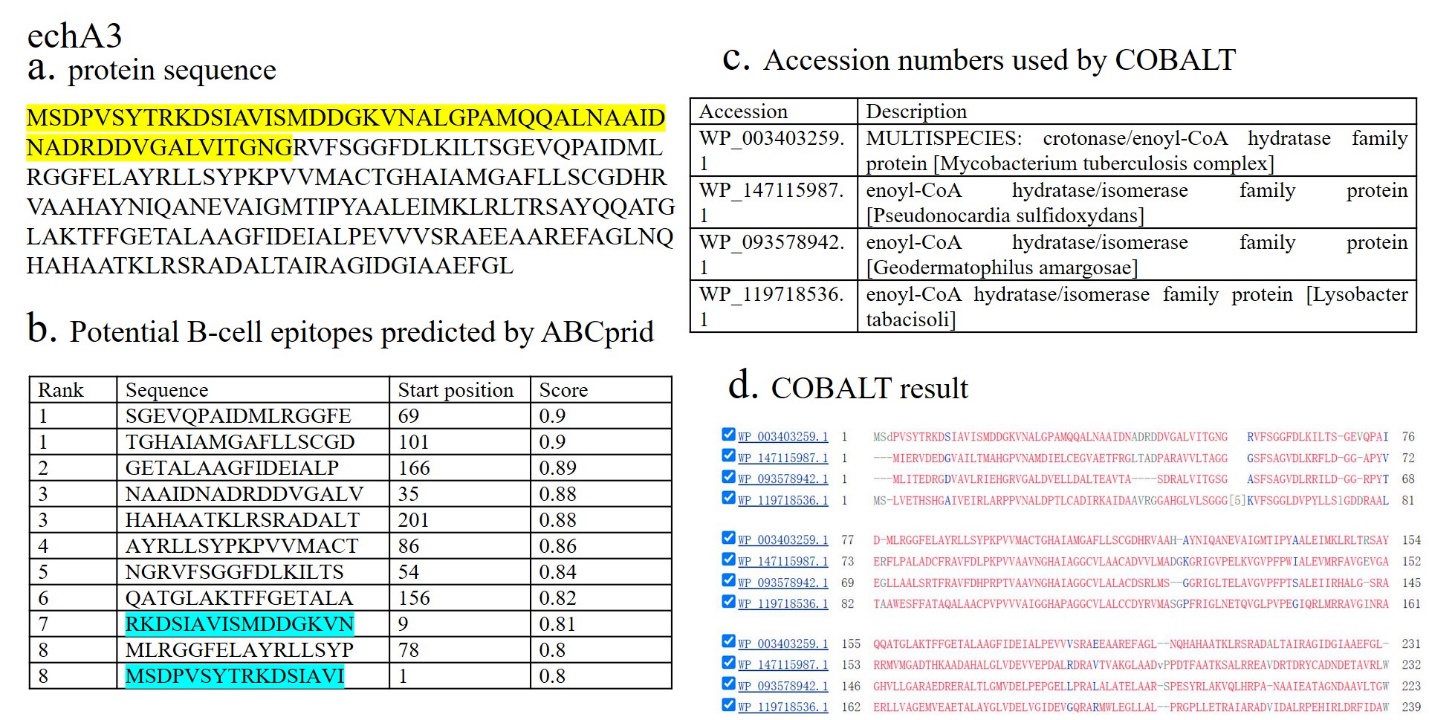


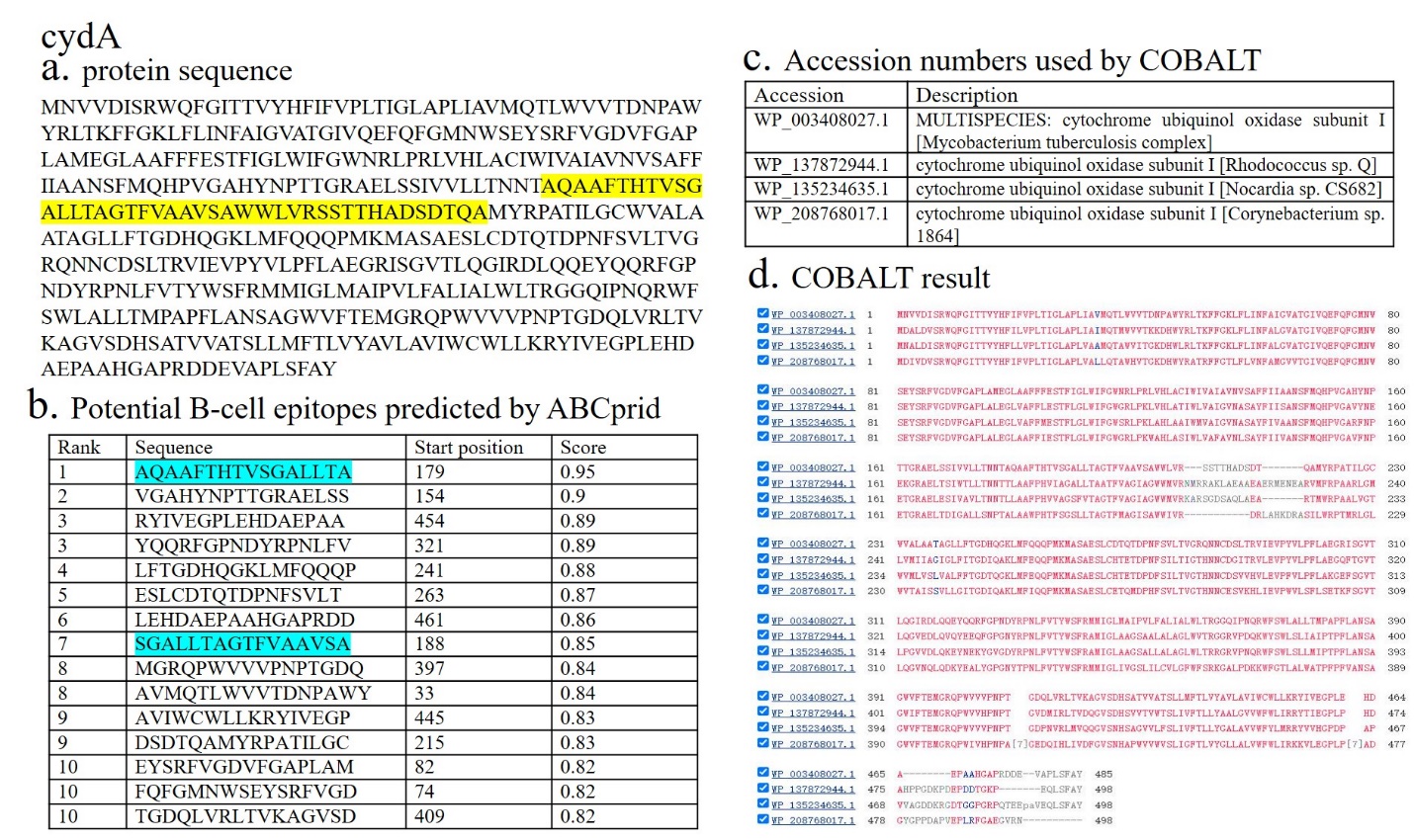


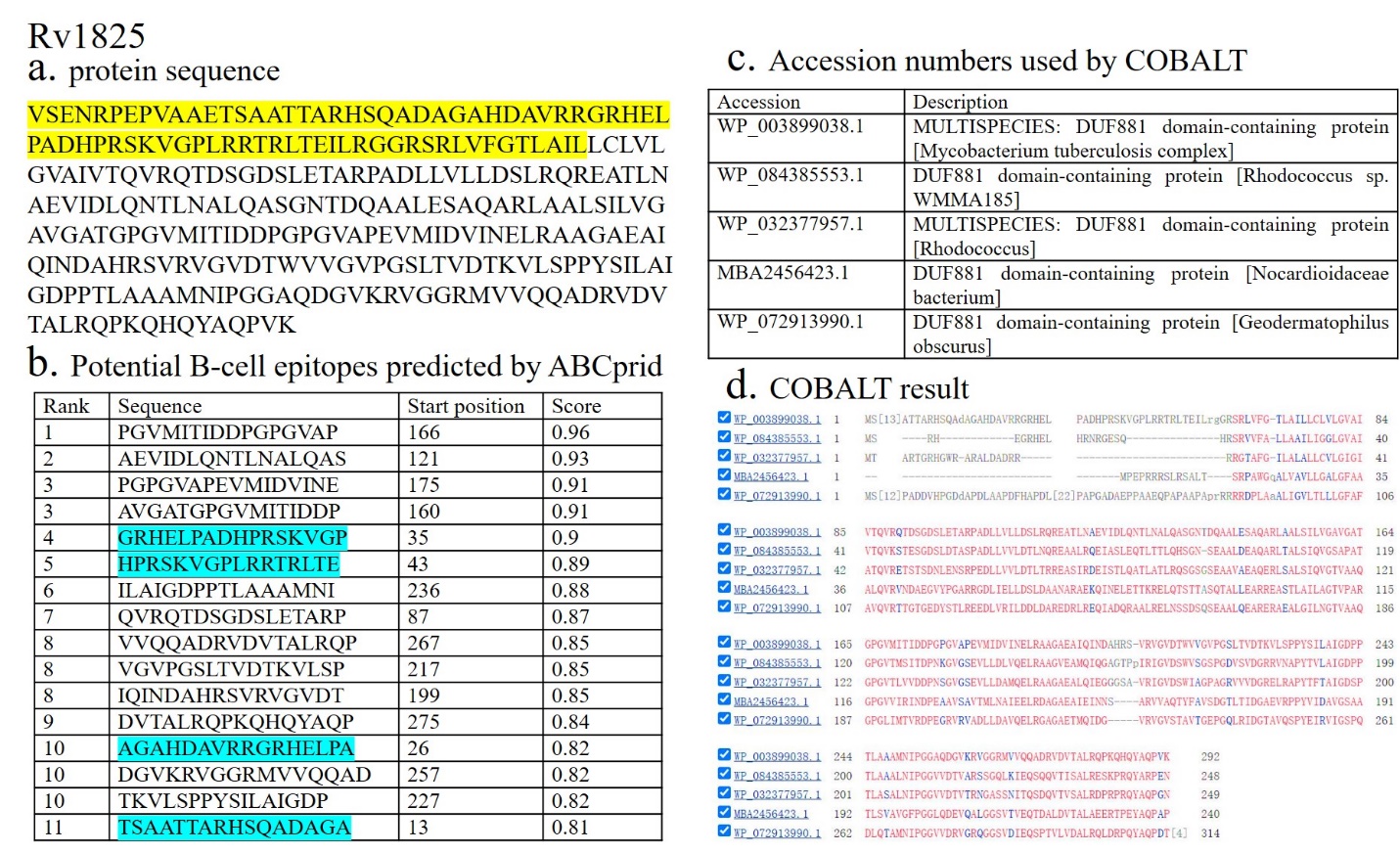


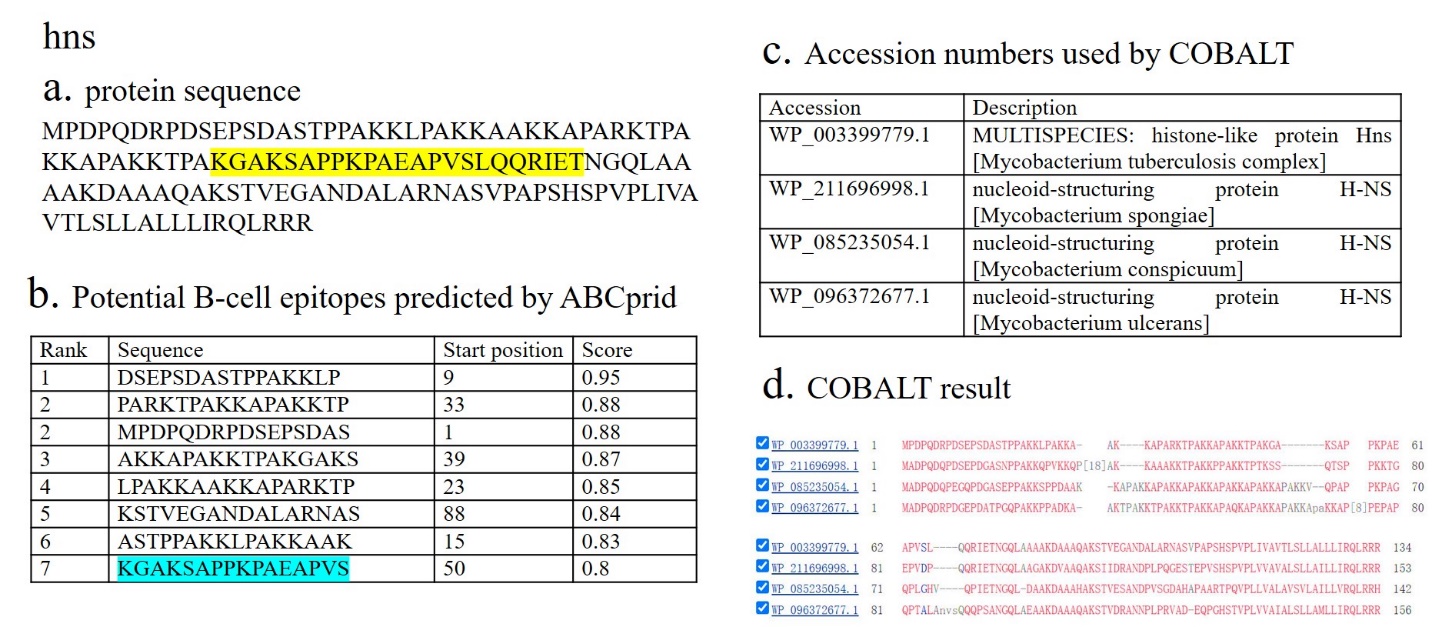


**Supplementary Figure 2**


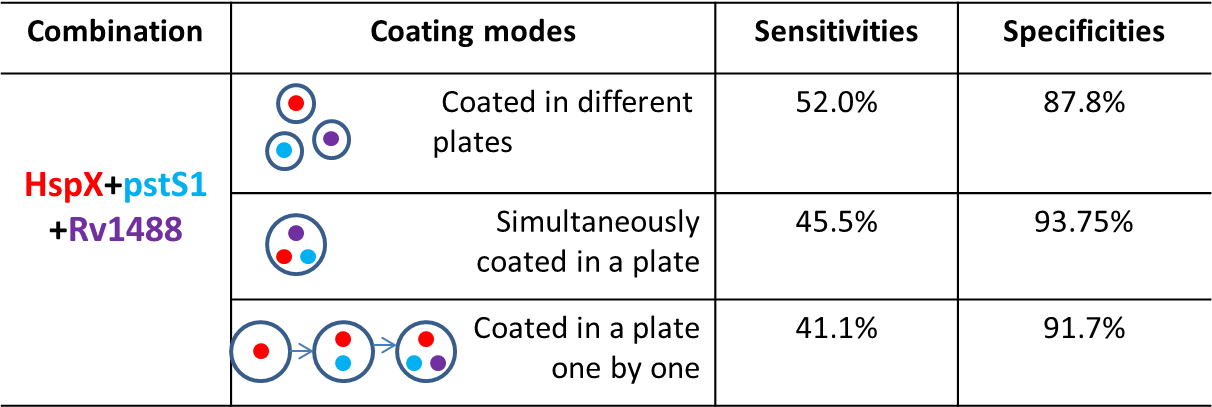


**Supplementary Figure 3**


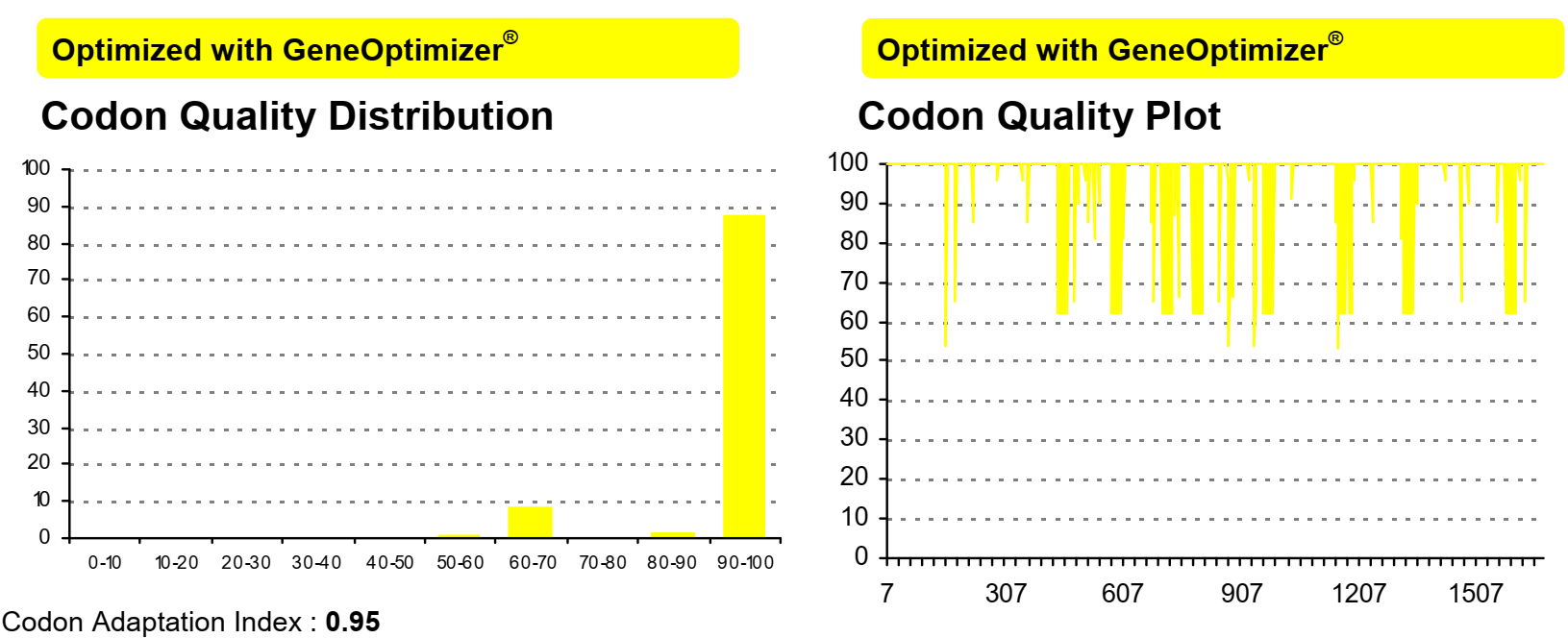

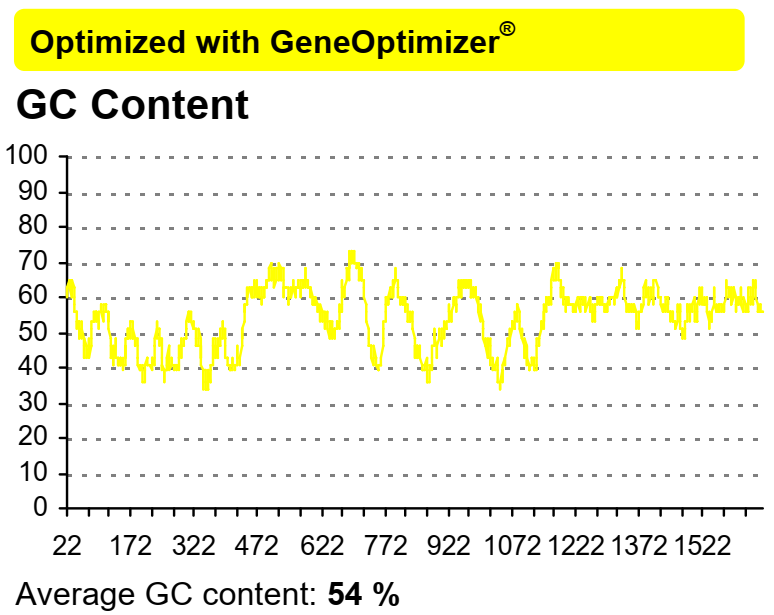


**Supplementary Figure 4**


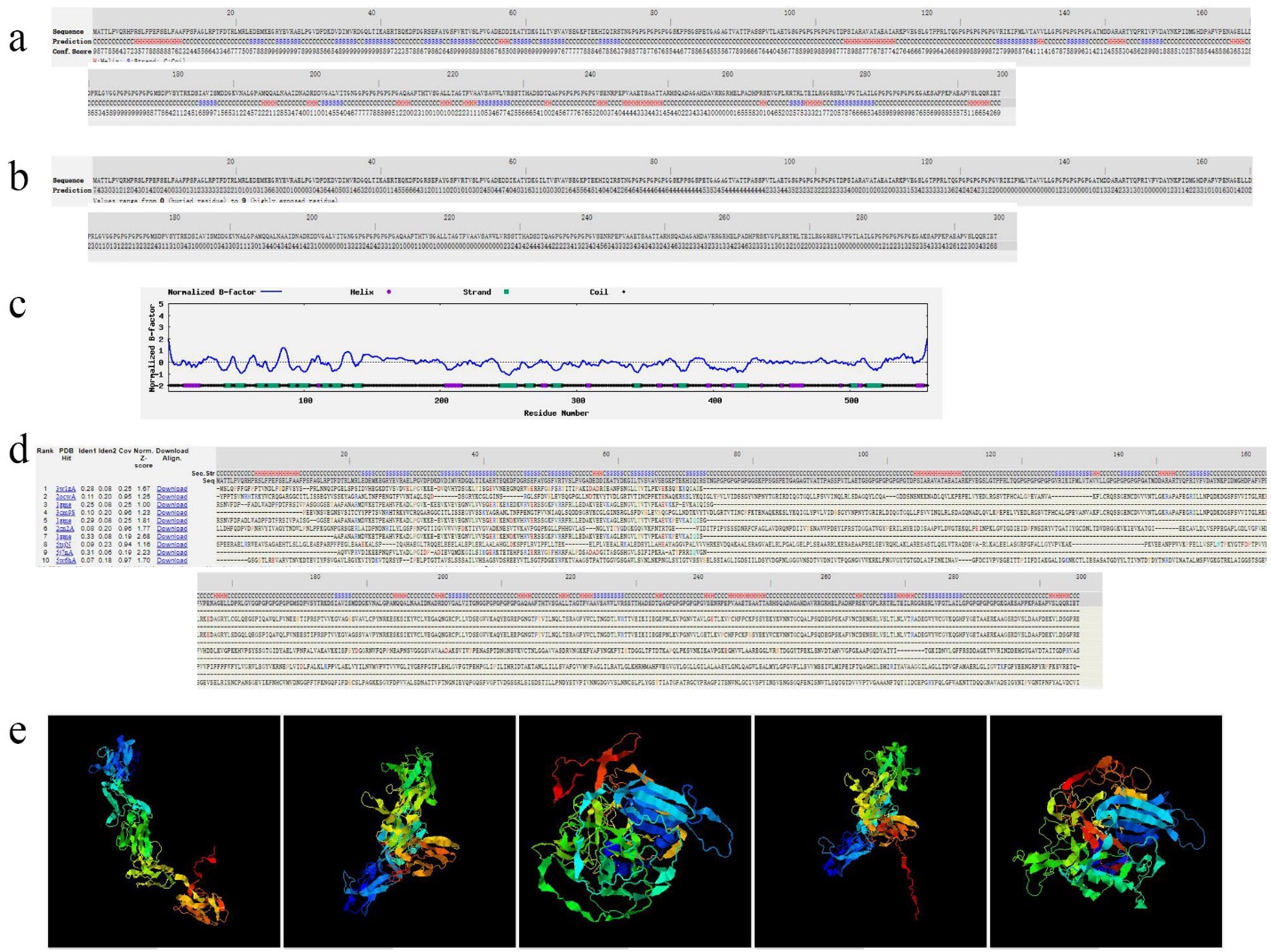


**Supplementary Figure 5**


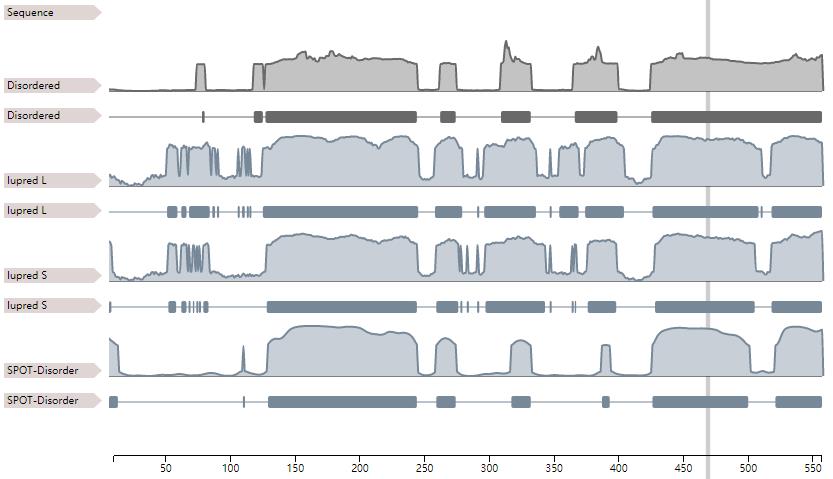


**Supplementary Figure 6**


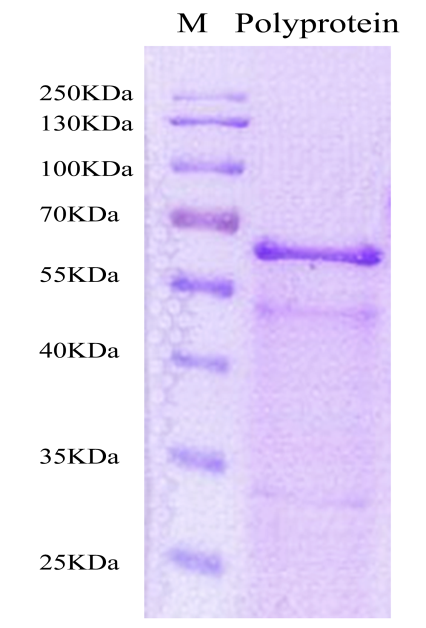


**Supplementary Table 1 Clinical characteristics of the study population.**

| **Characteristic** | **Active TB** | **Healthy control** |
| --- | --- | --- |
| **Round one** |  |  |
| Number | 72 | 24 |
| Age, median (range) (yr) | 39.5 (23-52) | 41.3 (33-57) |
| Male/female | 47/25 | 15/9 |
| sputum smear +/- | 32/40 | NA.^#^ |
| Chest X-ray +/- | 23/49 | NA. |
| **Round two** |  |  |
| Number | 63 | 33 |
| Age, median (range) (yr) | 35.8 (27-70) | 32.5 (18-71) |
| Male/female | 42/21 | 18/15 |
| sputum smear +/- | 28/35 | NA. |
| Chest X-ray +/- | 19/34 | NA. |
| **Round three** |  |  |
| Number | 128 | 64 |
| Age, median (range) (yr) | 33.8 (19-79) | 34.8 (23-62) |
| Male/female | 72/56 | 39/25 |
| sputum smear +/- | 49/79 | NA. |
| Chest X-ray +/- | 36/92 | NA. |
| **Round four** |  |  |
| Period of enrollment (m, yr) | August 2016 - December 2017 | |

^#^ Abbreviation: NA., not applicable

**Supplementary Table 2 Using lysates from various bacteria for sera pre-adsorption to reduce non-specific reactions (for Rv1488)**

| **Rv1488** | ***V. aquatilis*** | ***S. aureus*** | ***B. subtilis*** | ***S. epidermidis*** | ***P. vulgaris*** | ***E. aerogenes*** | ***γ-***  ***streptococcus*** | ***S. citreus*** | ***E.***  ***coli*** | **Mixed** | **BG** | **NC** |
| --- | --- | --- | --- | --- | --- | --- | --- | --- | --- | --- | --- | --- |
| **SFP** | 0.479 | 0.387 | 0.423 | 0.393 | 0.171 | 0.127 | 0.422 | 0.414 | 0.091 | 0.078 | 0.466 | 0.015 |
| **WFP** | 0.293 | 0.233 | 0.245 | 0.267 | 0.131 | 0.198 | 0.273 | 0.236 | 0.095 | 0.080 | 0.283 | 0.016 |
| **SP** | 0.748 | 0.622 | 0.631 | 0.613 | 0.515 | 0.594 | 0.649 | 0.623 | 0.447 | 0.147 | 0.786 | 0.027 |
| **HC** | 0.095 | 0.041 | 0.079 | 0.054 | 0.049 | 0.061 | 0.068 | 0.043 | 0.053 | 0.048 | 0.068 | 0.013 |

Abbreviation: Background, BG; Strong false positive, SFP; Weak false positive, WFP; strong positive, SP; Healthy control, HC; Negative control; *Vibrio aquatilis, V. aquatilis; Staphylococcus aureus, S. aureus; Bacillus subtilis, B. subtilis; Proteus vulgaris, P. vulgaris; Staphylococcus albus, S. epidermidis; Enterobacter aerogenes, E. aerogenes; gamma-streptococcus, γ-Streptococcus; Staphylococcus citreus, S. citreus; Escherichia coli, E.coli.* Noted: the measuring value is OD.

**Supplementary Table 3 Comparison of the effect of pre-adsorption by *P. vulgaris, E. aerogenes* and *E. coli (*for *Rv1488)***

| **Rv1488** | **BG** | ***E. coli*** | ***E. aerogenes*** | ***P. vulgaris*** | **Mixed** | **Blank** |
| --- | --- | --- | --- | --- | --- | --- |
| **FP1** | 0.913 | 0.270 | **0.707** | **0.700** | 0.294 | 0.013 |
| **FP2** | 1.075 | 0.271 | 0.296 | 0.272 | 0.196 | 0.009 |
| **FP3** | 0.670 | 0.268 | 0.293 | **0.558** | 0.242 | 0.007 |
| **FP4** | 0.305 | 0.209 | 0.177 | 0.222 | 0.166 | 0.008 |
| **FP5** | 0.566 | 0.337 | **0.451** | **0.495** | 0.345 | 0.009 |
| **FP6** | 0.360 | 0.174 | 0.202 | 0.191 | 0.153 | 0.016 |
| **FP7** | 1.497 | 0.359 | 0.468 | **0.826** | 0.381 | 0.014 |
| **FP8** | 0.407 | 0.193 | 0.248 | 0.222 | 0.219 | 0.011 |
| **FP9** | 0.481 | 0.217 | 0.201 | 0.192 | 0.177 | 0.007 |
| **FP10** | 1.203 | 0.180 | 0.222 | 0.184 | 0.147 | 0.007 |
| **FP11** | 0.645 | 0.163 | 0.176 | 0.187 | 0.155 | 0.007 |
| **FP12** | 0.434 | 0.196 | 0.201 | 0.214 | 0.210 | 0.009 |
| **P1** | 0.357 | 0.235 | 0.199 | 0.192 | 0.197 | 0.007 |
| **P2** | 0.705 | 0.171 | 0.363 | 0.196 | 0.147 | 0.009 |
| **HC1** | 0.159 | 0.147 | 0.144 | 0.135 | 0.089 | 0.009 |
| **HC2** | 0.092 | 0.054 | 0.077 | 0.094 | 0.047 | 0.007 |

Abbreviation: *Enterobacter aerogenes, E. aerogenes*; *Escherichia coli, E. coli*; Background, BG; False positive, FP; strong positive, SP; Healthy control, HC; Negative control. Noted: the measuring value is OD. Bold values indicated that the value of corresponding serum samples did not significantly decrease after adding bacteria lysates.

**Supplementary Table 4 Comparison of the effect of pre-adsorption by *E. coli* for the polyprotein and peptides**

| **Polyprotein** | BG | *E. coli* | **HspX** | BG | *E. coli* | **pstS1** | BG | *E. coli* | **Rv1488** | BG | *E. coli* |
| --- | --- | --- | --- | --- | --- | --- | --- | --- | --- | --- | --- |
| FP1-1 | 0.463 | **0.471** | FP1-2 | 0.595 | 0.471 | FP1-3 | 0.501 | 0.484 | FP1-4 | 0.327 | 0.223 |
| FP2-1 | 0.554 | 0.390 | FP2-2 | 0.575 | 0.392 | FP2-3 | 0.351 | 0.349 | FP2-4 | 0.478 | 0.471 |
| FP3-1 | 0.316 | 0.261 | FP3-2 | 0.401 | 0.268 | FP3-3 | 0.275 | 0.264 | FP3-4 | 0.229 | 0.182 |
| FP4-1 | 0.898 | 0.510 | FP4-2 | 0.469 | **0.514** | FP4-3 | 0.408 | **0.430** | FP4-4 | 0.633 | 0.465 |
| FP5-1 | 0.231 | 0.162 | FP5-2 | 0.546 | 0.365 | FP5-3 | 0.282 | **0.269** | FP5-4 | 0.449 | 0.320 |
| FP6-1 | 0.252 | 0.138 | FP6-2 | 0.417 | 0.237 | FP6-3 | 0.449 | 0.319 | FP6-4 | 0.484 | **0.412** |
| FP7-1 | 0.478 | 0.287 | FP7-2 | 0.227 | 0.166 | FP7-3 | 0.664 | 0.426 | FP7-4 | 0.266 | 0.194 |
| FP8-1 | 0.383 | 0.171 | FP8-2 | 0.260 | 0.153 | FP8-3 | 0.465 | 0.313 | FP8-4 | 0.517 | 0.294 |
| SP1-1 | 0.982 | 0.712 | SP1-2 | 1.290 | 1.042 | SP1-3 | 0.582 | 0.296 | SP1-4 | 1.107 | 0.861 |
| SP2-1 | 0.464 | 0.333 | SP2-2 | 0.360 | 0.306 | SP2-3 | 0.303 | 0.234 | SP2-4 | 0.278 | 0.230 |
| HC1-1 | 0.121 | 0.091 | HC1-2 | 0.057 | 0.060 | HC1-3 | 0.072 | 0.054 | HC1-4 | 0.077 | 0.082 |
| HC2-1 | 0.075 | 0.080 | HC2-2 | 0.065 | 0.111 | HC2-3 | 0.048 | 0.083 | HC2-4 | 0.102 | 0.075 |
| Reduction (%) | 87.5% (7/8) | | Reduction (%) | 87.5% (7/8) | | Reduction (%) | 75.0% (6/8) | | Reduction (%) | 87.5% (7/8) | |
| **mpt64** | BG | *E. coli* | **panD** | BG | *E. coli* | **echA3** | BG | *E. coli* | **cydA** | BG | *E. coli* |
| FP1-5 | 0.200 | 0.110 | FP1-6 | 0.271 | 0.170 | FP1-7 | 0.415 | **0.379** | FP1-8 | 0.255 | 0.143 |
| FP2-5 | 0.178 | 0.090 | FP2-6 | 0.384 | 0.190 | FP2-7 | 0.292 | 0.125 | FP2-8 | 0.197 | 0.093 |
| FP3-5 | 0.179 | **0.160** | FP3-6 | 0.193 | 0.160 | FP3-7 | 0.161 | 0.116 | FP3-8 | 0.442 | 0.231 |
| FP4-5 | 0.145 | 0.110 | FP4-6 | 0.223 | **0.210** | FP4-7 | 0.249 | 0.177 | FP4-8 | 0.178 | 0.140 |
| FP5-5 | 0.166 | 0.160 | FP5-6 | 0.190 | 0.160 | FP5-7 | 0.179 | 0.139 | FP5-8 | 0.257 | 0.165 |
| FP6-5 | 0.377 | **0.330** | FP6-6 | 0.239 | 0.130 | FP6-7 | 0.378 | 0.171 | FP6-8 | 0.367 | 0.125 |
| FP7-5 | 0.239 | 0.150 | FP7-6 | 0.266 | **0.280** | FP7-7 | 0.197 | 0.087 | FP7-8 | 0.194 | **0.165** |
| FP8-5 | 0.271 | 0.170 | FP8-6 | 0.241 | 0.150 | FP8-7 | 0.253 | **0.222** | SFP8-8 | 0.162 | 0.067 |
| SP1-5 | 0.187 | 0.150 | SP1-6 | 0.181 | 0.160 | SP1-7 | 0.533 | 0.484 | SP1-8 | 0.313 | 0.243 |
| SP2-5 | 0.482 | 0.380 | SP2-6 | 0.422 | 0.330 | SP2-7 | 0.449 | 0.405 | SP2-8 | 0.534 | 0.393 |
| HC1-5 | 0.024 | 0.029 | HC1-6 | 0.072 | 0.090 | HC1-7 | 0.022 | 0.046 | HC1-8 | 0.077 | 0.085 |
| HC2 | 0.035 | 0.040 | HC2-6 | 0.091 | 0.080 | HC2-7 | 0.011 | 0.032 | HC2-8 | 0.016 | 0.026 |
| Reduction (%) | 75.0% (6/8) | | Reduction (%) | 75.0% (6/8) | | Reduction (%) | 75.0% (6/8) | | Reduction (%) | 87.5% (7/8) | |
| **Rv1825** | BG | *E. coli* | **hns** | BG | *E. coli* |  |  |  |  |  |  |
| FP1-9 | 0.777 | 0.497 | FP1-10 | 0.274 | **0.213** |  |  |  |  |  |  |
| FP2-9 | 0.203 | 0.110 | FP2-10 | 0.187 | **0.164** |  |  |  |  |  |  |
| FP3-9 | 0.198 | 0.093 | FP3-10 | 0.392 | 0.184 |  |  |  |  |  |  |
| FP4-9 | 0.192 | 0.101 | FP4-10 | 0.176 | 0.106 |  |  |  |  |  |  |
| FP5-9 | 0.336 | **0.268** | FP5-10 | 0.294 | 0.140 |  |  |  |  |  |  |
| FP6-9 | 0.233 | 0.166 | FP6-10 | 0.197 | **0.205** |  |  |  |  |  |  |
| FP7-9 | 0.191 | 0.095 | FP7-10 | 0.168 | 0.115 |  |  |  |  |  |  |
| FP8-9 | 0.264 | 0.153 | FP8-10 | 0.193 | 0.103 |  |  |  |  |  |  |
| SP1-9 | 0.653 | 0.579 | SP1-10 | 0.501 | 0.432 |  |  |  |  |  |  |
| SP2-9 | 0.459 | 0.361 | SP2-10 | 0.468 | 0.388 |  |  |  |  |  |  |
| HC1-9 | 0.060 | 0.035 | HC1-10 | 0.036 | 0.020 |  |  |  |  |  |  |
| HC2-9 | 0.046 | 0.037 | HC2-10 | 0.045 | 0.055 |  |  |  |  |  |  |
| Reduction (%) | 87.5% (7/8) | | Reduction (%) | 62.5% (5/8) | |  |  |  |  |  |  |

Abbreviation: *Escherichia coli, E. coli*; Background, BG; False positive, FP; strong positive, SP; Healthy control, HC; Negative control. Noted: the measuring value is OD. Bold values indicated that the value of corresponding serum samples did not significantly decrease after adding bacteria lysates.

**Supplementary Table 5 Predicted polypeptides of the fusion polyprotein**

| **Rv.** | **Gene** | **Genebank**  **Accession** | **Amio acid sequence** | **No.** |
| --- | --- | --- | --- | --- |
| Rv2031c | HspX | CCP44804.1 | MATTLPVQRHPRSLFPEFSELFAAFPSFAGLRPTFDTRLMRLEDEMKEGRYEVRAELPGVDPDKDVDIMVRDGQLTIKAERTEQKDFDGRSEFAYGSFVRTVSLPVGADEDDIKATYDKGILTVSVAVSEGKPTEKHIQIRSTN | 144 |
| Rv0934 | pstS1 | CCP43682.1 | GSKPPSGSPETGAGAGTVATTPASSPVTLAETGS | 34 |
| Rv1488 | - | NP_216004.1 | TDPSIARAVATAEAIARKPVEGSLGTPPRLTQ | 32 |
| Rv1980c | mpt64 | CCP44749.1 | VRIKIFMLVTAVVLL | 15 |
| Rv3601c | panD | CCP46424.1 | ATMDDARARTYQPRIVFVDAYNKPIDMGHDPAFVPENAGELLDPRLGVG | 49 |
| Rv0632c | echA3 | NP_215146.1 | MSDPVSYTRKDSIAVISMDDGKVNALGPAMQQALNAAIDNADRDDVGALVITGNG | 55 |
| Rv1623c | cydA | YP_177824.1 | AQAAFTHTVSGALLTAGTFVAAVSAWWLVRSSTTHADSDTQA | 42 |
| Rv1825 | - | NP_216341.1 | VSENRPEPVAAETSAATTARHSQADAGAHDAVRRGRHELPADHPRSKVGPLRRTRLTEILRGGRSRLVFGTLAIL | 75 |
| Rv3852 | hns | CCP46681.1 | KGAKSAPPKPAEAPVSLQQRIET | 23 |

**Supplementary Table 7 Comparison of reactivity of the polyprotein in different populations**

| Subgroups | Positive | Negative | Sensitivity (%, 95% CI) | P Value |
| --- | --- | --- | --- | --- |
| Sputum smear (+) | 31 | 18 | 63.3(48.3–76.6) | 0.57 |
| Sputum smear (+) | 46 | 33 | 58.2(46.6–69.2) |  |
| Chest X-ray (+) | 20 | 16 | 55.6(38.1–72.1) | 0.51 |
| Chest X-ray (-) | 57 | 35 | 62.0(51.2–71.9) |  |

Data was analyzed by the Chi-square test by SPSS.
